# A combined experimental and modeling approach to elucidate the adsorption mechanism for sustainable water treatment via In_2_S_3_-anchored chitosan

**DOI:** 10.1038/s41598-023-45506-4

**Published:** 2023-10-23

**Authors:** Soumya Ranjan Mishra, Prerona Roy, Vishal Gadore, Md. Ahmaruzzaman

**Affiliations:** Department of Chemistry, National Institute of Technology, Silchar, India

**Keywords:** Pollution remediation, Nanoscale materials

## Abstract

A novel Chitosan/Indium sulfide (CS/In_2_S_3_) nanocomposite was created by co-precipitating Chitosan and InCl_3_ in solution, resulting in In_2_S_3_ agglomeration on the Chitosan matrix with a remarkable pore diameter of 170.384 Å, and characterized it for the physical and chemical properties. Under optimal conditions (pH = 7, time = 60 min, catalyst dosage = 0.24 g L^−1^, and dye concentration = 100 mg L^-1^), the synthesized nanocomposite demonstrated remarkable adsorption capabilities for Victoria Blue (VB), attaining a removal efficiency of 90.81%. The Sips adsorption isotherm best matched the adsorption process, which followed pseudo-second-order kinetics. With a rate constant of 6.357 × 10^–3^ g mg^−1^ min^−1^, the highest adsorption capacity (q_m_) was found to be 683.34 mg g^−1^. Statistical physics modeling (SPM) of the adsorption process revealed multi-interaction and multi-molecular adsorption of VB on the CS/In_2_S_3_ surface. The nanocomposite demonstrated improved stability and recyclability, indicating the possibility for low-cost, reusable wastewater dye removal adsorbents. These results have the potential to have practical applications in environmental remediation.

## Introduction

Water contamination is a significant environmental hazard that has gained global concern. Both human life and the natural environment have been significantly affected by the same due to the swift decrease in the availability of freshwater resources^1,2^. Various pollutants contaminate water, including organic dyes, phenolic compounds, heavy metal ions, inorganic ions, radioactive wastes, and other pharmaceutical contaminants. Among these, organic dyes are the most hazardous and toxic due to their inherent mutagenicity and carcinogenicity^3,4^. These dyes also degrade very slowly due to their relatively complex and stable structures; hence, they persist and accumulate in the aquatic environment over a long time^5^. Such dyes harm human health and aquatic life by entering the food chain and depleting the hygienic standard of the water it persists in. Therefore, wastewater treatment has become the need of the hour and an essential strategy for a sustainable future^6,7^.

**Figure 1:**
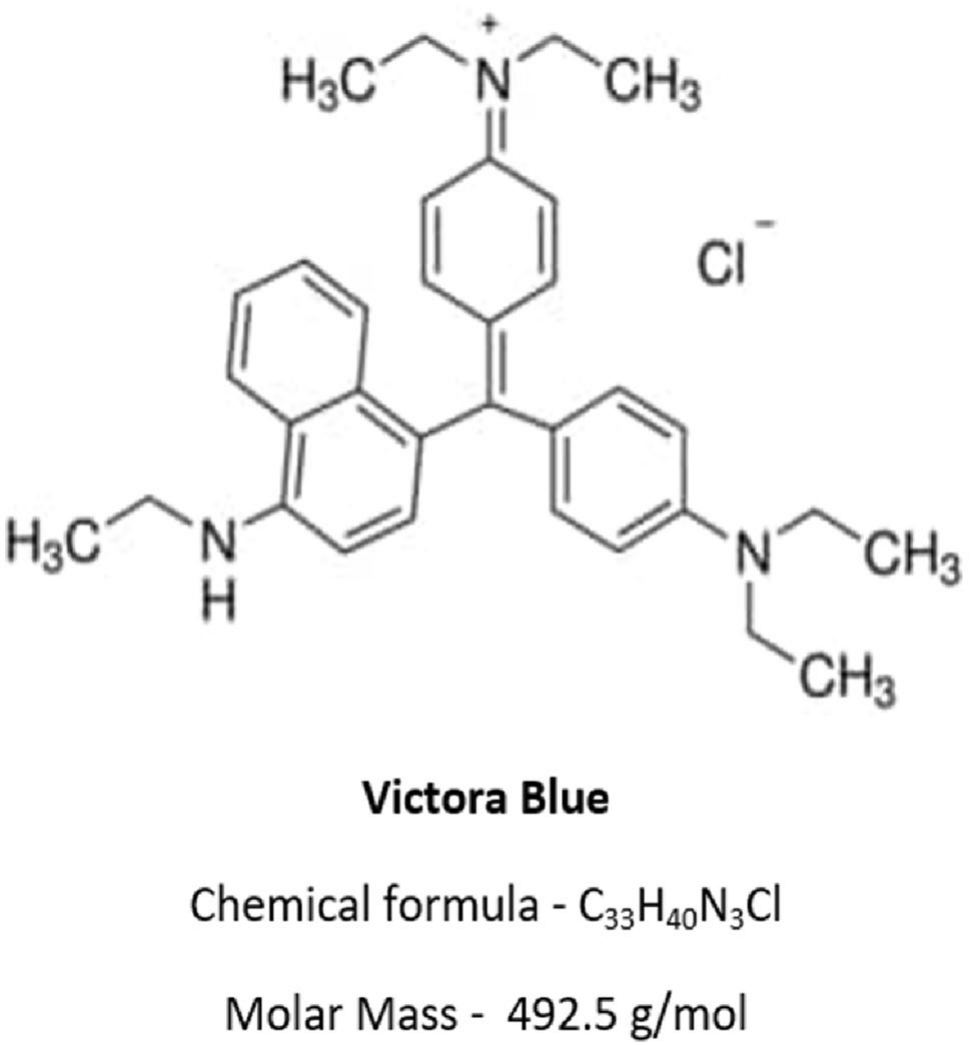
Chemical Structure of VB.

Victoria Blue (VB) is a dye that can cause detrimental effects on aquatic ecology and affect human well-being in various ways (Fig. 1). VB is a cationic blue dye (M.W. = 492.5 g/mol) that is biodegradable in nature. It is a brown powder melting at 206 °C, which dissolves in water to form a dark blue solution. It is broadly utilized for dyeing cotton, wool, and silk, as well as for staining and pigmentation of microscopic specimens. Moreover, it acts as a photosensitizer, inducing cytotoxic responses in various mammalian cell linings^8^. It is found to cause eye irritation and, in some cases, permanent opacification on long exposures to human eyes. Other than this, it is also reported to cause fatal diseases such as pneumoconiosis (a lung disease) and skin injuries on prolonged exposure^9^. The decision to investigate this basic dye was motivated by the apparent scarcity of studies on removing this specific VB dye using CS and metal sulfide. Conventional methods for wastewater treatment can be of three types: biological, chemical, and physical. These methods include membrane separation^10^, adsorption^11^, advanced oxidation^12,13^, catalytic ozonation^14^, Fenton and electro-Fenton^15^, catalytic photodegradation^16–18^, electrochemical degradation^19^, ultrasonic irradiation^20^ and biodegradation^21^. Adsorption has gained widescale popularity due to its easy operations, cost-effectiveness, eco-friendly nature, and broad applicability^22^. Adsorption depends on various physicochemical factors, like the surface area of the adsorbent, pH of the solution, operating temperature, and the characteristics of the adsorbate^23^. In this vein, scientists are continuously developing various functional adsorbents for improved elimination of pollutants from an aqueous solution.

Generally, separating the used adsorbent from the treated sample is a typical task. Due to the difficulty in the separation of nanoparticles, several support materials are used to enhance adsorption efficiency. Another reason to use supporting materials is that they prevent the agglomeration of metal sulfide nanoparticles and increase the prepared nanocomposite's surface area, thereby enhancing the adsorption capacity^24^. The properties possessed by an ideal adsorbent include abundant availability of active sites or more considerable surface exposure, high saturation capacity, appropriate pore volume and pore size, convenient accessibility, abundance, selectivity towards the target contaminant, pocket, and environment-friendly nature. Hence, scientists are developing various adsorbents using natural products such as biopolymers as they tend to have higher adsorption capacities and are both environmentally friendly and cost-effective^25^. Biopolymers have been one of the most efficient adsorbent materials worldwide. These have been used effectively in large-scale industrial and large-scale industrial and manufacturing applications for wastewater treatment, food production, drug delivery, biomedical purposes, chemical engineering, and fuel cell production^26^. Among various biopolymers, Chitosan is one of the most inexpensive, non-toxic, available, renewable, biodegradable, and biocompatible amino-polysaccharide^27^. Alkaline deacetylation of the naturally available fiber chitin leads to the creation of chitosan^28^. Perhaps the most prevalent biopolymer in the environment, chitin, is derived from the shells of crustaceans, including prawns, crabs, and lobsters, which are leftovers from the seafood business. Specific properties that make chitosan an efficient adsorbent include its macromolecular structure, cationic, availability, low-cost, eco-friendly nature, and interactive amino and hydroxyl functionalities^29^. It has been described as possessing sturdy inter and intra-molecular hydrogen bonds. However, the strong covalent bonds of chitosan that run along the backbone of the biopolymer chain due to strong hydrogen bonding make it difficult for dye molecules to access its active sites and restrict its solubility, reactivity, and adsorption capability^30^. As an amino polysaccharide, chitosan gains its stable and feasible structure and adsorption efficiency from the existing interactive amino and hydroxyl functionalities^31^. The high amino content of the polymer facilitates various chemical and physical alterations that improve the polymer's adsorption capabilities and adsorption capacity.

Two two-dimensional metal sulfides have recently attracted researchers worldwide for their advanced surface-to-volume ratio, facile synthesis, and tunable surface properties. A wide range of semiconductor nanomaterials has also shown splendid adsorption performances. Various metal sulfides have been synthesized to remove organic contaminants from wastewater. Indium sulfide is a semiconductor with unique thermal, optoelectrical, mechanical, and physical properties^32^. It has a bandgap range from 2.0 eV to 2.4 eV^33^. These are widely used in photodetectors, solar cells, photocatalytic degradation, and biological imaging^34^. Indium sulfide is widely known for its photocatalytic dye removal from wastewater, and its adsorption potential is still a rising topic for worldwide research. Researchers recently fabricated In_2_S_3_/g-C_3_N_4_ nanocomposite via a hydrothermal method for mercury adsorption^35^. Flower-like β- In_2_S_3_ was designed to adsorb MB dye^36^. In addition, we have also developed β-In_2_S_3_/BC for the effective adsorption of methylene blue and malachite green from single and binary systems^37^.

This literature aims to fabricate and study the application of CS/In_2_S_3_ nanocomposite in wastewater treatment by removing VB (λ_max_ = 616 nm) from the contaminated water. The fabricated nanocomposite catalyst has the advantage of the existing interactive functionalities of the biopolymer, such as the NH_2_ and OH groups on the large surface area of the In_2_S_3_ particles, which enhances the adsorption capacity of the biopolymer towards the target contaminant. The nanocomposite showed appreciable adsorption capacity and removal efficiency towards VB. The developed Chitosan/In_2_S_3_ nanocomposite showed an exceptionally high q_m_ of 683.34 mg g^−1^ with an outstanding removal efficiency of 90.81% at room temperature, higher than pristine In_2_S_3_ nanoparticles. The adsorption procedure obeyed the Sips isotherm model, and the adsorption mechanism was studied using statistical physics modeling (SPM). The interaction between the dye and the adsorbent surface may be used to calculate the experimental adsorption isotherm. The evaluation of isotherm statistics is critical to comprehending the process governing adsorption. The function of the functional groups in the sorption of VB can be examined and tested by fusing SPM calculations with the characterization and adsorption observational findings^38^. Furthermore, data fitting via well-known adsorption isotherm models (such as Langmuir, Sips, Freundlich, and Redlich-Peterson) can result in solid conclusions for maximal adsorption capacity and affinity. However, they do not offer crucial details for elucidating adsorption mechanisms or pertinent information on adsorption dynamics. The mechanism of dye adsorption has been examined using statistical physics models in order to close this knowledge gap. The crystalline composition and topography of the fabricated nanocomposite were extensively studied. This article highlights the fabrication, characterization, application, isotherm, kinetics, thermodynamics, physical modeling, the role of the adsorption parameters and inorganic and organic compounds, water matrices, mechanism, and reusability of the CS/In_2_S_3_ nanocomposite.

## Experimentation

### Reagents

Chitosan, Indium chloride tetrahydrate (InCl_3_.4H_2_O), Sodium sulfide nonahydrate (Na_2_S.9H_2_O), Sodium dodecyl sulfate (NaC_12_H_25_SO_4_), HCl, Victoria blue (VB), and ethanol. The aforementioned compounds were obtained from Sigma Aldrich. Since they are of analytical quality, they need not be further purified before usage. Throughout this experiment, de-ionized water is utilized in several procedures.

Although adsorption research is geared toward ecological purposes, the majority of analyses are conducted in de-ionized water. Few studies have investigated the impact of natural water samples on organic contaminants' adsorption activities. An analysis of the influence of various inorganic ions and organic compounds was conducted to gain a better insight into the nanocomposite's effectiveness and remove contaminants from different water samples, such as bottled mineral, tap, and lake water. For this study, packaged bottled mineral water was bought from a nearby shop. Water for the lake was obtained from lakes inside the National Institute of Technology, Silchar, in the Indian state of Assam, which served as the source for the water matrix analysis. Tap water was made available through the neighborhood drinking water systems.

### Sample preparation

#### *Fabrication of In*_*2*_*S*_*3*_* nanoparticle*

Pristine In_2_S_3_ nanoparticle was synthesized using the co-precipitation method, as carried out by Kennedy et al.^39^. 2 mmol of Indium chloride tetrahydrate (InCl_3_.4H_2_O) and 0.1 mmol of Sodium dodecyl sulfate (NaC_12_H_25_SO_4_) was taken in a beaker and stirred at 500 rpm for about 30 min at room temperature. After that, 3 mmol of Sodium sulfide nonahydrate (Na_2_S.9H_2_O) solution was introduced dropwise into the above solution, and the mixture was agitated at 1200 rpm for two h at 60 °C. As a result, a bright yellow precipitate was obtained, which was rinsed with de-ionized water and ethanol, and dried at 65 °C until the final product was obtained.

#### *Fabrication of CS/In*_*2*_*S*_*3*_* nanocomposite*

The CS/In_2_S_3_ adsorbent was also produced through the simple co-precipitation technique, following the work of Gadore et al.^40^. Chitosan was added to 72 mL of 1 M HCl solution and agitated at 1000 rpm for 1 h under moderate heat (Solution A). Simultaneously, indium chloride tetrahydrate (InCl_3_.4H_2_O) was dissolved in 50 mL of de-ionized water and mixed at 1000 rpm for 15 min (Solution B). CS and InCl_3_.4H_2_O were taken in a 1:1 weight ratio. After that, solution B was mixed with solution A, and the resulting solution (Solution C) was heated and agitated at 1200 rpm for 1 h. 1.5 mmol sodium sulfide nonahydrate (Na_2_S.9H_2_O) solution was added dropwise to solution C with constant mixing and moderate heating. The final solution was kept under magnetic stirring for 1 h, and the resulting precipitate was centrifuged, washed with ethanol and de-ionized water, and dried at 65 °C till the final product was obtained.

### Characterization of fabricated CS/In_2_S_3_

Throughout the work, various analytical methods were used to characterize both In_2_S_3_ and the composite material CS/In_2_S_3_. X-ray diffraction (XRD) was carried out using Philips X'PERT equipment, Cu-K radiation, and a scanning rate of 2° per minute at room temperature. We also used Transmission electron microscopy (TEM) with a JEOL JEM 2100 apparatus to examine the diffraction patterns of CS/In_2_S_3_, concentrating on characterizing diffraction ring forms, diameters, and size distribution. SEM and Elemental mapping was carried out on an FEI Quanta FEG 200-High Resolution Scanning Electron Microscope to analyze the surface characteristics, elemental distribution, and composition of the produced nanocomposite. The sample's elemental composition was carefully determined using energy-dispersive X-ray (EDX) analysis. The Brunauer–Emmett–Teller (BET) technique was used to calculate the specific surface area of the materials using the Quantachrome Novae 2200 instrument. Furthermore, an X-ray Photoelectron Spectroscopy (XPS) investigation was performed on the materials using a Thermafischer scientific Nexsa base system to get insight into their chemical composition and bonding states. To record the absorbance spectrum, UV–visible (UV–Vis) spectroscopic data were obtained using a GENESYS 10S UV–visible spectrophotometer with a quartz cuvette with a path length of 1 cm and a wavelength scan speed of 600 nm per minute. These scientific procedures enabled a complete and exact assessment of the materials under consideration.

### Adsorption analysis

The sorption tests were carried out in dark conditions at a temperature of nearly 298 K. Fluctuating dye concentrations and catalyst dosages optimized the adsorption effectiveness of the generated adsorbent. 6–14 mg of the adsorbent were added to a 50 mL mixture containing 100 ppm VB to maximize the catalyst dosage. The mixture that emerged was swirled in the dark for 60 min. 2 mL of the resulting mixture was then transferred to a test tube and centrifuged. The effectiveness of the adsorbent was ascertained by gauging the absorbance of the subsequently separated mixture with a UV–visible spectrophotometer at an λ_max_ of 616 nm. The optimal dye concentration was then determined by introducing the optimized catalyst quantity to a range of VB dye concentrations (100^−^160 mg L^−1^). The dye concentration and catalyst dosage were optimized to evaluate the adsorption capacity (q_e_), contact time, and impact on adsorption due to different organic and inorganic substances and water matrices. In order to analyze the adsorption isotherms again, the optimal dosage of the nanocomposite was added to beakers containing 50 mL of 10–400 mg L^−1^ VB at a varied temperature of 298–398 K, and the resultant solution was stirred in the dark for 60 min. The following Eqs. (1) and (2) can be utilized to obtain the adsorption capacity and removal efficacy.

1 $$ Efficiency\% = \frac{{C_{0} - C_{t} }}{{C_{0} }} \times 100 $$

2 $$ q_{e} = \frac{{C_{0} - C_{e} }}{m} \times V $$

Where V is the total volume of the solution (mL), m represents the mass of the adsorbent or nanocomposite (mg), C_0_, C_t,_ and C_e_ are the concentrations of the dye solution time at the initial stage, at time t, and equilibrium (mg L^−1^), and q_e_ is the maximum adsorption capacity of the nanocomposite (mg g^−1^).

### Statistical physics modeling to elucidate the dye removal mechanism

Organic pollutants like VB can be absorbed by creating one or more adsorption layers on the adsorbent. As a result, two distinct statistical physics models that considered the creation of mono and multiple adsorption layers, which clarified the dye adsorption mechanism, were utilized to fit the investigational isotherm calculations. For the experiment, dye solutions of 10–400 mg L^−1^. were taken and were adsorbed at temperatures from 298 to 328 K for an equilibrium time. The obtained non-linear curve was fitted to the model to acquire the desired parameters.

## Results and discussions

### Characterization of the prepared nanocomposite

CS/In_2_S_3_ nanocomposite was synthesized via an in-situ growth procedure employing the co-precipitation technique. Firstly, Chitosan was dissolved in HCl, followed by indium chloride, adding In^3+^ ions to incorporate into the chitosan matrix. Secondly, the CS/In_2_S_3_ was precipitated by adding sodium sulfide (Na_2_S), forming In_2_S_3_ on the chitosan matrix. The fabricated nanocomposite was characterized to establish the structure, morphology, composition, and topography.

#### X-ray diffraction analysis

**Figure 2:**
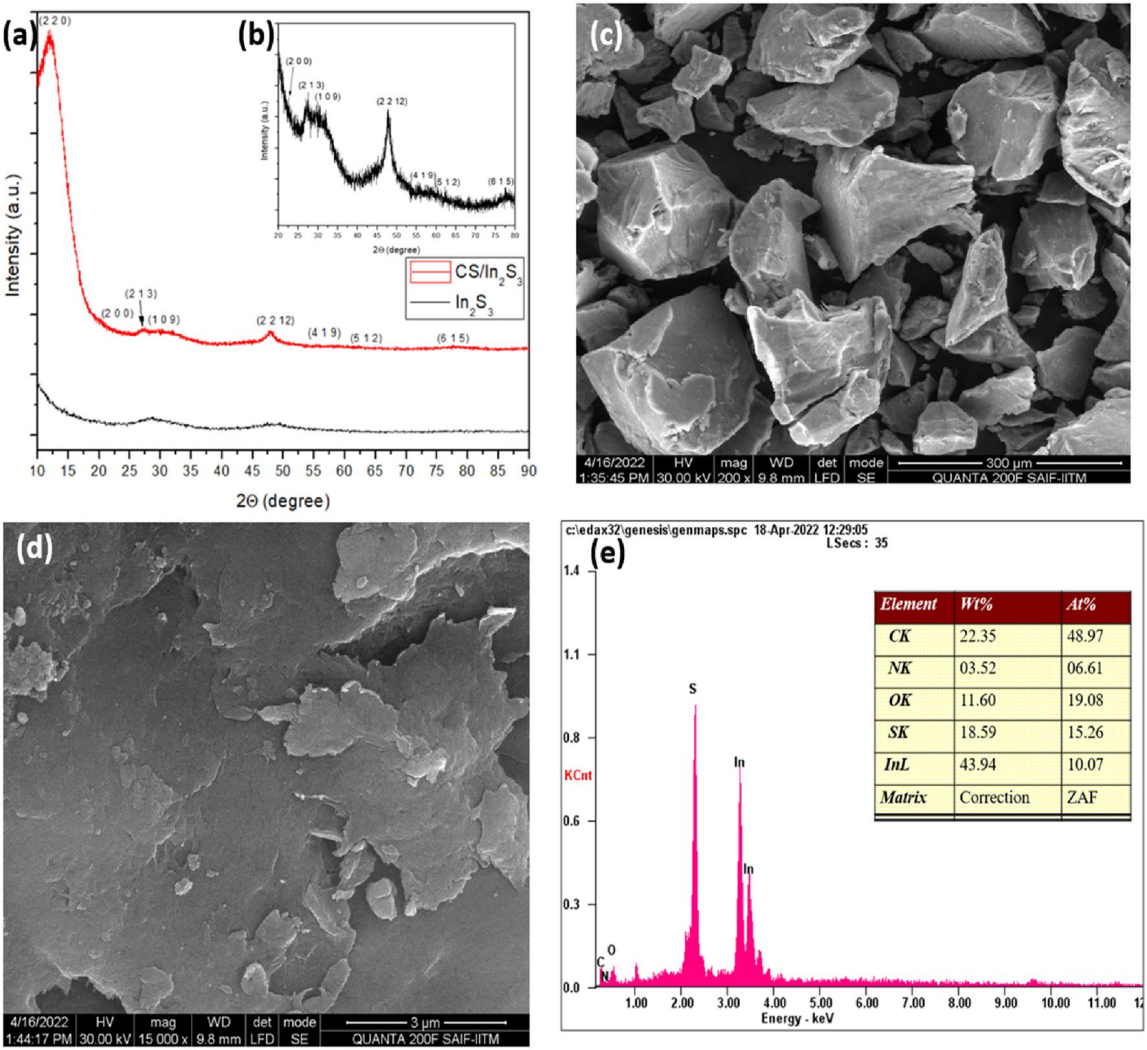
XRD patterns of (**a**) In_2_S_3_ and CS/In_2_S_3_; (**b**) magnified peak of CS/In_2_S_3_; (**c**, **d**) FE-SEM images, (**e**) EDX analysis of fabricated CS/In_2_S_3_ (peak table inserted).

The structure of the CS/In_2_S_3_ nanocomposite was first characterized by XRD to determine the phase composition and structural and chemical features of the fabricated nanocomposite. The XRD plot of the fabricated nanomaterial and nanocomposite and the magnified XRD plot of the nanocomposite are displayed in Fig. 2a,b, correspondingly. The XRD plot in Fig. 2a shows one intense and distinct crystalline peak for chitosan polymer at 2θ = 12.19° indexed to the reflection plane (2 2 0). Previous studies revealed the mean intermolecular distance of the crystalline components of chitosan and discovered that intramolecular and intermolecular hydrogen bonds are principally accountable for chitosan's firm crystalline structure^41^. In the XRD plot of pure CS, the amorphous phase of the polymer is represented by two broad peaks centered at 2θ = 55° and 35°^42^. However, in the XRD plot of the nanohybrid, it is observed that the intensity of two broad peaks has remarkably reduced; this might be owed to the interaction between the metal sulfide and Chitosan. Therefore, it can be inferred that the formation of the nanocomposite has relatively reduced the amorphous nature of the biopolymer. The XRD data of In_2_S_3_, as depicted in Fig. 2a,b, showed numerous intense and distinct Bragg's reflection peaks. The peaks displaying the angle 2θ = 23.39^0^, 27.54^0^, 29.61^0^, 47.72^0^, 55.80^0^, 62.21^0^ and 77.53^0^ can be indexed to the (2 0 0), (2 1 3), (1 0 9), (2 2 12), (4 1 9), (5 1 2) and (6 1 5) reflections of tetragonal In_2_S_3_ (associated with JCPDS Card No. 73–1366) owning space group I41/amd (141), lattice parameters a = 7.623 Å and c = 32.360 Å^43,44^. It is fascinating to observe that analogous XRD patterns were detected in CS/In_2_S_3_ (Fig. 2b), indicating that the indium sulfide developed on the surface of Chitosan grew in (2 2 12) and (1 0 9) directions as the relative diffraction peaks of these lattice planes are relatively high^43^. The average size of the crystallite is obtained from Debye–Scherrer's equation (Eq. 3):

3 $$ D = \frac{k\lambda }{{\beta \cos \theta }} $$

where D is the crystallite's size in nanometers (nm), k is its form factor (0.89), λ is the wavelength of CuKα1 radiation (1.54060 Å), is a peak's full width at half maximum (FWHM), and θ is the Bragg's angle of diffraction. The crystallite size D of In_2_S_3_ is ~ 9.602 nm.

#### Scanning electron microscope and EDX analysis

Figure 2c,d demonstrate the FE-SEM images of the synthesized nanocomposite prepared by developing Indium sulfide on the surface of HCl-treated chitosan. The nanocomposites are irregular in shape, and as seen in Fig. 2c, the chitosan surface is relatively uneven. Moreover, some fractures are observed on the surface due to the nanocomposite formation. Thus, it can be concluded that the nanocomposite surface becomes coarse and flake-like due to the grafting of In_2_S_3_ and chitosan biopolymer^45^. The uneven and porous structure enhanced the adsorption of dye molecules even further^46^. The distribution of elements in the synthesized CS/In_2_S_3_ was inspected additionally by EDX and portrayed in Fig. 2e. The developed nanocomposite is found to have carbon, oxygen, sulfur, nitrogen, and indium elements in the composite structure, with a weight percentage of 22.35, 11.60, 18.59, 3.52, and 43.94%, respectively. As revealed in Fig. 2e, the atomic percentage suggests that indium and sulfur are present in a 2:3 ratio. Chitosan's persistence in the composite hybrid matrix accounts for the nanocomposite's high atomic percentages of carbon, oxygen, and nitrogen^46^.

#### Transmission electron microscope analysis

**Figure 3:**
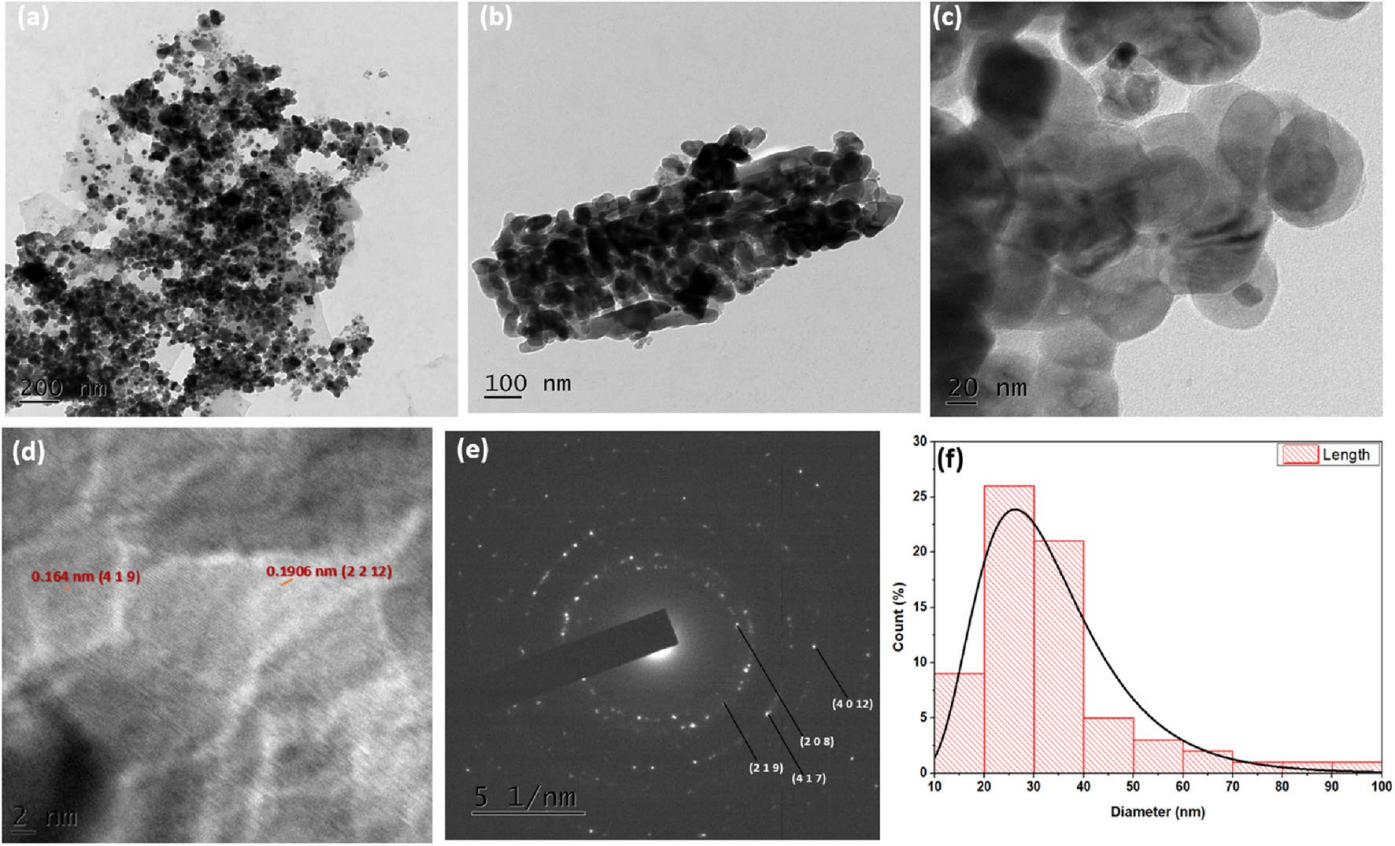
(**a**–**e**) TEM analysis of the prepared CS/In_2_S_3_ composite; (**f**) Graph displaying mean particle size of the synthesized CS/In_2_S_3_ composite.

The CS/In_2_S_3_ nanocomposite's structural characteristics were analyzed through a transmission electron microscope (TEM). The TEM pictures in Fig. 3a,b depict the non-uniform dispersion of the nanocomposite. The HR-TEM image (Fig. 3c) revealed the accumulation of the In_2_S_3_ nanoparticles over the polymeric matrix of CS. The average crystal diameter of CS/In_2_S_3_ was determined quantitatively using a histogram fitted with Lorentzian function and was calculated to be 26.23 nm (Fig. 3f). The HR-TEM (Fig. 3d) exhibited interplanar lattice spacing of 0.1906 nm and 0.164 nm, which agrees to the (2 2 12) and (4 1 9) planes of In_2_S_3_. The non-uniform distribution of intensity along the perimeters of the visible concentric rings depicted in Fig. 3e reveals the polycrystalline nature of the fabricated nanocomposite^47^. Also, as evident from the XRD plot in Fig. 2b, the amorphous nature of the biopolymer is reduced on agglomerating metal sulfides on its surface; hence, its crystalline nature is more predominant^48^. In Fig. 3e, the diameters of four characteristic circles are given as 7.223, 8.331, 11.641, and 12.270 nm, which correspond to the d-spacing of 2.7740, 2.400, 1.718, and 1.556 nm indexed to (2 0 8), (2 1 9), (4 1 7), and (4 0 12) planes of In_2_S_3_ respectively.

#### X-ray photoelectron spectroscopy analysis

**Figure 4:**
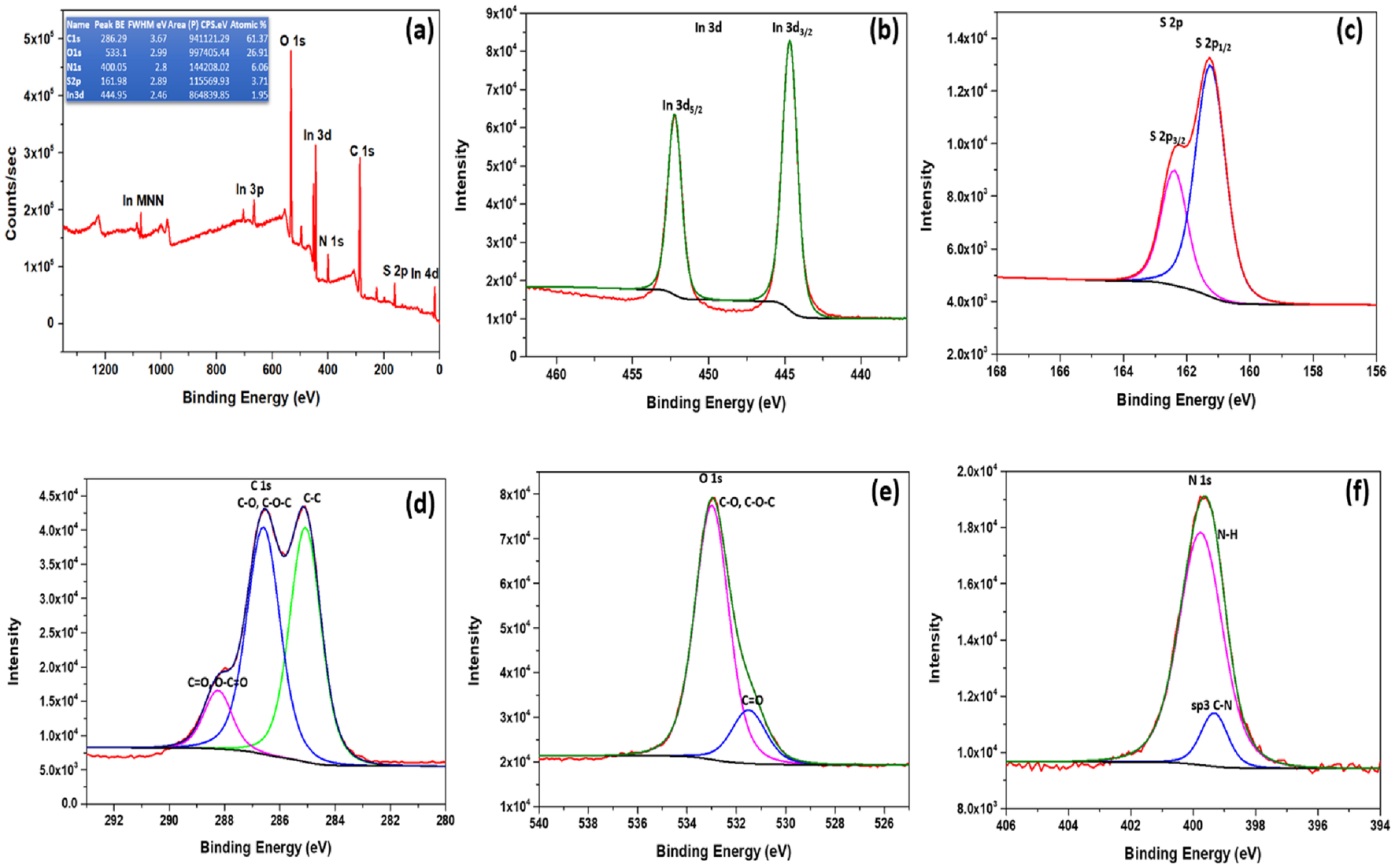
(**a**) Survey spectrum of synthesized CS/In_2_S_3_ (peak table incorporated). XPS short scan spectrum of (**b**) In3d, (**c**) S2p, (**d**) C1s, (**e**) O1s, (**f**) N1s.

X-ray photoelectron spectroscopy (XPS) was used to analyze the CS/In_2_S_3_ nanocomposite's chemical composition. The chemical status and elemental makeup of the CS/In_2_S_3_ nano adsorbent were revealed by the findings of the XPS survey spectrum, as displayed in Fig. 4a. The survey spectrum showed the distinct peaks of In, S, C, O, and N, with little extra peaks indicating some impurity. Two unique peaks were visible in the HR spectra for In 3d (Fig. 4b), which is due to In 3d_5/2_ and In 3d_3/2_, correspondingly representing the + 3 oxidation state of In^49^. The crests for S 2p are exhibited in Fig. 4c at energies of 161.32 eV and 162.27 eV, accordingly, which correspond to S's 2p_1/2_ and 2p_3/2_ sulfide anions and terminal polysulfide^50^. It demonstrates that the component that directly bonds In^3+^ to the chitosan moiety is sulfur. Inferring In_2_S_3'_s existence from this is likewise possible. In the XPS spectrum for carbon C 1 s, as illustrated by Fig. 4d, three distinct peaks are observed at 285.09 eV, 286.29 eV, and 288.51 eV; these show the presence of C–C, C–O–C, and C-O, and C = O and O–C = O bond present in the chitosan matrix correspondingly^51^. The peak for O 1 s (Fig. 4e) shows a distinct rise at 533.1 eV, which depicts a shift from the original value of 530 eV, indicating the formation of the nanohybrid along with presence of C–O–H, C–O–C, and N–C = O bonds in chitosan^51^. Figure 4f illustrates the XPS spectrum for N 1 s, and it shows peaks at 399.35 eV and 399.80 eV, showing the sp^3^–C–N bond and N–H bonds present in the base polymer of the nanohybrid, respectively^52^.

#### Elemental mapping and brunauer–emmett–teller analysis

**Figure 5:**
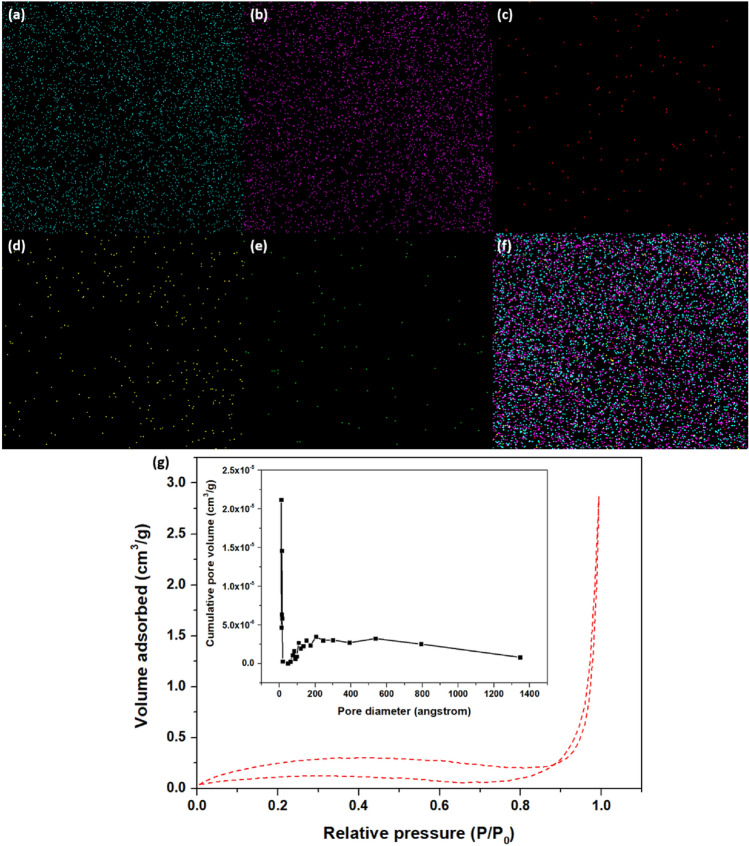
Elemental mapping images of (**a**) Indium, (**b**) sulfur, (**c**) Carbon, (**d**) Oxygen, (**e**) Nitrogen, and (**f**)overall elements for CS/In_2_S_3_ nanocomposite; (**g**) N_2_ adsorption–desorption curve for BET analysis and BJH pore distribution curve (fitted inside).

We can infer from Fig. 5 that In_2_S_3_ nanoparticles are uniformly dispersed over the chitosan surface. The elemental mapping images from Fig. 5a–f show an even dispersal of In, S, C, O, and N in CS/In_2_S_3_. The CS/In_2_S_3_ nanocomposite was also characterized by employing the Brunauer–Emmett–Teller (BET) analysis. The nanocomposite's specific surface area was calculated to be 10.0494 m^2^/g, while its pore volume and diameter were found to be 0.004470 cm^3^/g and 170.384 Å, respectively. The pore diameter size shows the existence of mesopores on the catalyst’s surface, inset of Fig. 5g, as it usually ranges between 20 and 500 Å^53^. Thus, the high porosity of the material led to high dye adsorption and increased removal efficiency. Furthermore, the shape of the BJH distribution curve might reveal information on the connectivity of the pores inside the material. A wide distribution of linked holes may allow for improved fluid flow, resulting in remarkable adsorption capability by the manufactured adsorbent. Also, the BET constant c and the q_m_ were obtained as 17.306906 and 0.2411 cm^3^/g at STP. The correlation of the coefficient of BET isotherm is 0.9988473. The N_2_ adsorption–desorption curve is depicted in Fig. 5g. We can observe the H1 type of hysteresis curve and type IV BET adsorption isotherm. BET type IV isotherm shows initial monolayer adsorption preceding multilayer adsorption. After the initial monolayer creation, the quantity adsorbed gradually increases in the adsorption isotherm, suggesting the production of multilayers of dye molecules. This is common in porous materials with varying pore diameters^54^. The mesoporous structure of the nanocomposite is further demonstrated by the hysteresis loop^55^. The results suggest that the adsorbent engages in chemisorption because of its reduced BET surface area and pore diameter but significantly higher pore volume.

### Adsorption studies of the prepared nanocomposite

VB dye was designated as a typical contaminant to study the adsorption capacity of the prepared CS/In_2_S_3_ composite. The adsorption isotherm, kinetics, and impact of catalyst doses, dye concentrations, and contact time were studied. As adsorption is temperature-dependent, the influence of temperature upon the removal of the model pollutant was investigated as well, and the thermodynamic variables were calculated. For the appraisal of the adsorbent, the consequence of multiple variables, such as the proportion of inorganic ions and organic compounds and the effect of different water matrices on the adsorption capacity of the prepared adsorbent, in addition to its reusability, was investigated.

#### Effect of catalyst dosage and dye concentration

**Figure 6:**
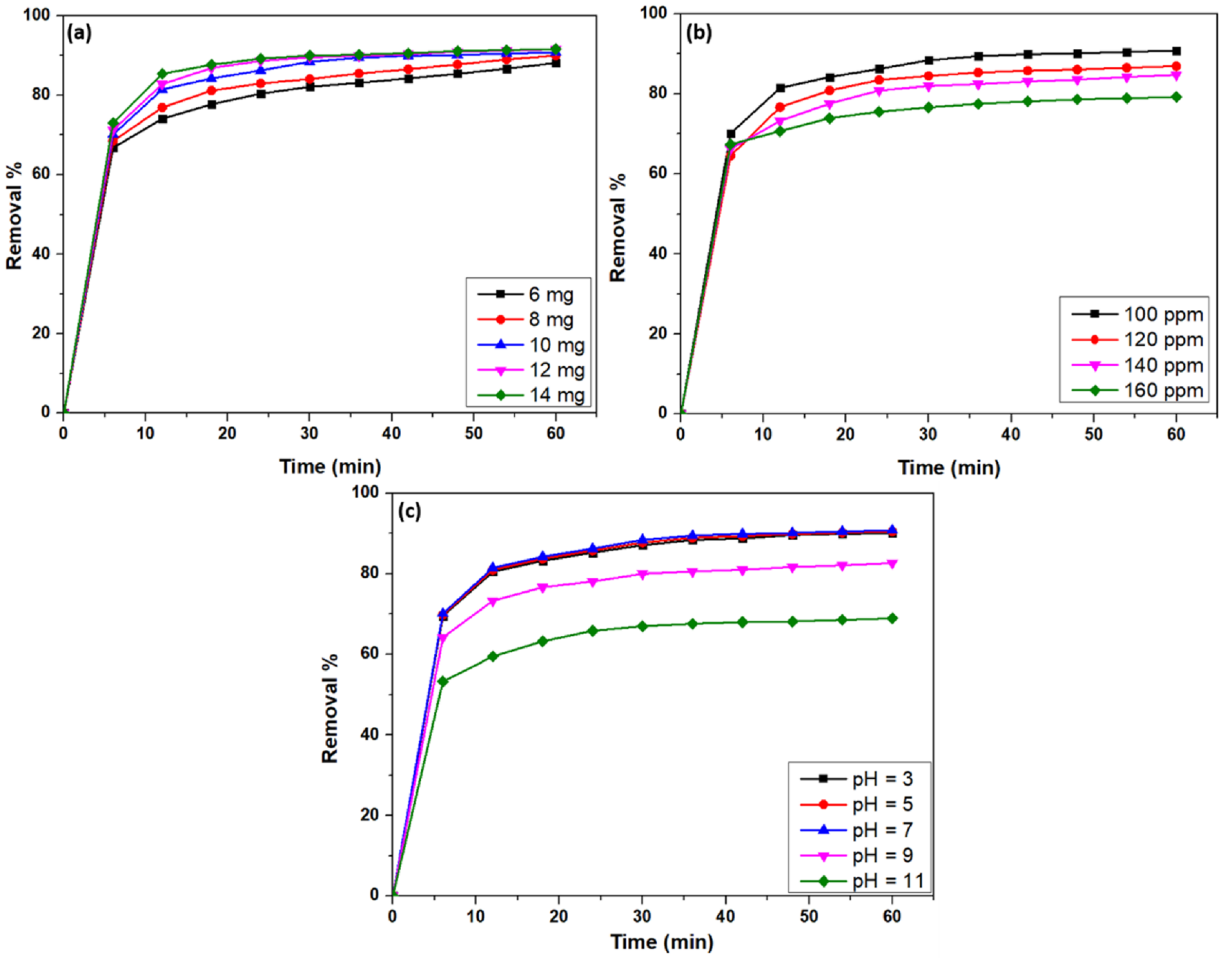
Removal percentage due to (**a**) variable catalyst dosage, (**b**) variable dye concentration, and (**c**) variable pH.

The amount of solid nanocomposite is critical to determine the removal percentage of the VB dye. 100 ppm of dye was taken, varying the catalyst dose to 6–14 mg (0.12 g L^−1^–0.28 g L^−1^) at room temperature, and was kept for 60 min in the dark. It was found that the 12 and 14 mg doses of the fabricated nanocomposite showed the highest removal percentage of about 90.81% (Fig. 6a). However, the optimum amount is considered to be 12 mg as it displayed an adsorption capacity (q_t_) of 279.32 mg L^−1^. In contrast, the 14 mg catalyst exhibited a q_t_ of 226.28 mg L^−1^. Furthermore, a specific mass of adsorbent can only adsorb a certain amount of the solute, making the starting concentration of the adsorbate crucial^56^. The batch adsorption was led to study the removal of VB dye, varying its concentration from 100 to 160 ppm, keeping the catalyst dose constant at 12 mg. The studies revealed that 100 ppm dye concentration showed the highest removal percentage of 90.81%, as shown in Fig. 6b. Therefore, for further studies, such as the influence of contact time, water matrix, the role of organic and inorganic compounds, and reusability, the optimized catalyst dose of 12 mg and dye concentration of 100 ppm was considered.

#### Effect of pH

This study looked at how pH affected the adsorption of VB onto a composite made of chitosan and In_2_S_3_. This thorough investigation involves altering pH values between 3 and 11. It was interesting to see that the results showed that the adsorption effectiveness of VB had a definite pH-dependent trend, with the best removal efficiency attained at pH 7, reaching an astounding 90.81% (Fig. 6c). The electrostatic interactions and surface charge characteristics of the CS/In_2_S_3_ composite material are responsible for this observed trend. The material's surface may have a positive charge under more acidic (lower pH) conditions, encouraging electrostatic interaction with the negatively charged VB molecules^57^. The surface, however, becomes less positively charged as the pH rises, potentially lowering the adsorption capability. The best removal effectiveness is likely achieved at a pH of 7, which strikes a balance between advantageous electrostatic interactions and changes in the charge state of the VB molecule^58^. These findings have significant significance for maximizing the usage of this composite material in water treatment applications and offer insightful information about the pH-dependent adsorption behavior of VB on CS/In_2_S_3_.

#### Effect of contact time

**Figure 7:**
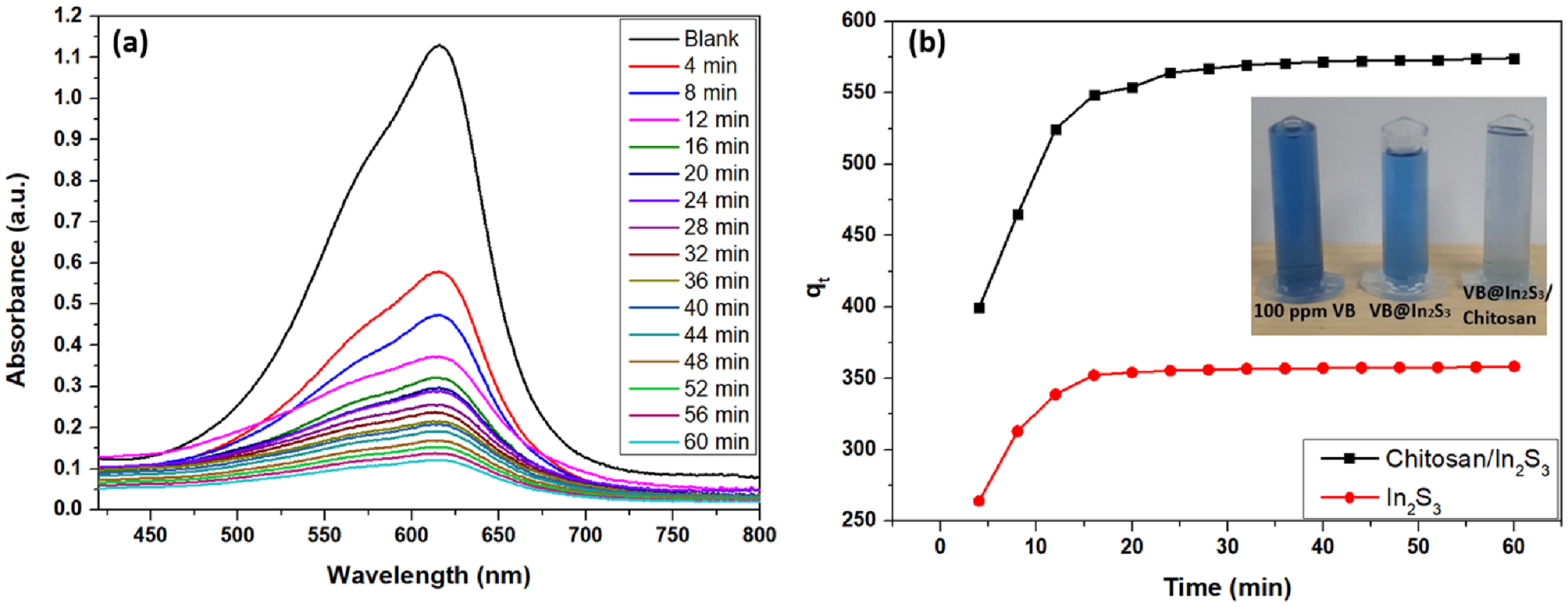
(**a**) Role of contact time on the adsorption of VB; (**b**) Influence on q_t_ with increasing time.

Essentially, this research aims to find the optimal environment for maximal adsorption by considering the contact duration between the liquid and solid^59^. The decrease in absorbance due to the decline in concentration is illustrated in Fig. 7a. The VB dye adsorption vs. contact time for a fixed amount of 12 mg of adsorbent demonstrates identical sudden rises at initial times before plateauing, as illustrated in Fig. 7b.

#### Adsorption isotherm

**Figure 8:**
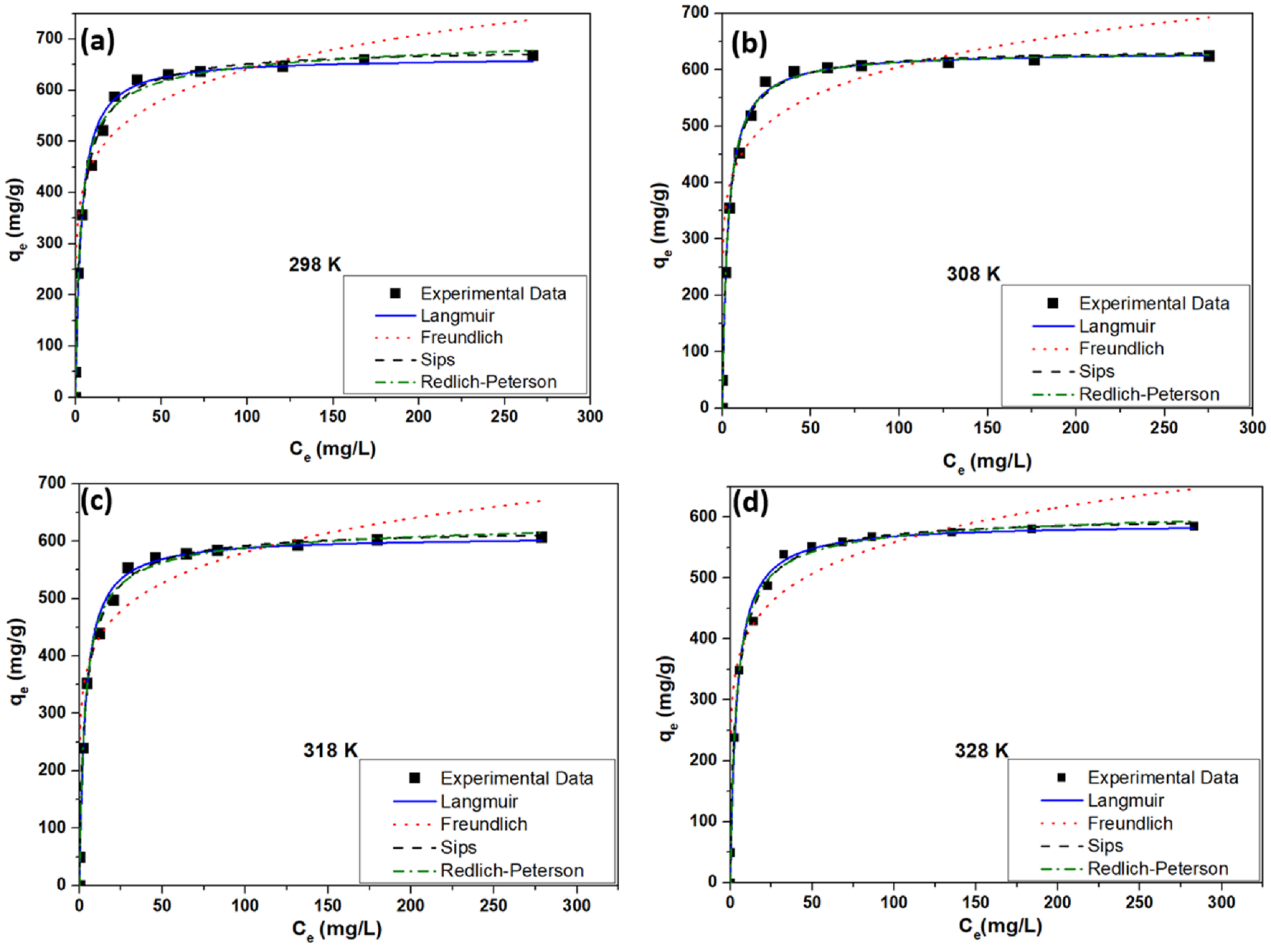
Adsorption isotherm models at varying temperatures.

Equilibrium isotherms define how dye molecules interact with the adsorbent and determine the maximal adsorption capacity (q_m_)^60^. The q_m_ of the fabricated adsorbent can be predicted by analyzing isotherm data, which constitutes one of the critical variables needed for designing adsorption systems^61^. The isotherm evaluations were conducted with variable initial concentrations of VB (10–400 mg L^−1^) at 298^−^328 K and 12 mg of the fabricated nanocomposite. Langmuir^62^, Freundlich^63^, Sips^64^, and Redlich-Peterson^65^ isotherm models (Fig. 8) were prepared, and it was inferred that the adsorption of VB obeyed the Sips isotherm model, which had the highest correlation coefficient (R^2^) of 0.9931, as demonstrated in Table 1. The sips isotherm model is a widely used adsorption model and is considered to be a combination of Langmuir and Freundlich isotherms. The q_m_ of the fabricated CS/In_2_S_3_ nanocomposite for VB was found to be 683.34 mg g^−1^ with a Sips constant of 0.0982 L mg^−1^. Both the monolayer and multilayer adsorption approaches follow the adsorption procedure. Values for 1/n (known as heterogeneity factor) in sips isotherm, near zero, indicate heterogeneous adsorbents, whereas values nearer to or 1.0 suggest the substance with relatively homogenous binding sites. 1/n value near 1 indicates the shifting of the isotherm to the Langmuir model; however, in this case, the values remain close to 0.5 at nearly all temperatures, indicating the co-existence of the monolayer and multi-layer adsorption process. Moreover, this inference was also backed by the BET adsorption–desorption curve, which followed type II adsorption that indicated monolayer adsorption followed by multilayer adsorption. Table 2 displays that the prepared nanocomposite exhibited a higher q_m_ for VB than the formerly testified research works.

**Table 1:** Comparison of various parameters by different isotherms.

Isotherm Models	Equations	Parameters	Temperature (Kelvin)			
			298 K	308 K	318 K	328 K
Langmuir	$q_{e} = \frac{{q_{m}^{L} bC_{e} }}{{1 + bC_{e} }}$	q_m_ b R^2^	664.66 0.3141 0.9855	632.69 0.3173 0.9929	608.48 0.2871 0.9906	598.71 0.2634 0.9904
Freundlich	$q_{e} = K_{F} C_{e}^{1/n}$	K_F_ 1/n R^2^	330.18 0.144 0.8227	327.52 0.133 0.7675	305.44 0.1395 0.8151	293.85 0.1396 0.8117
Sips	$q_{e} = \frac{{q_{m}^{s} (K_{S} C_{e} )^{1/n} }}{{1 + (K_{S} C_{e} )^{1/n} }}$	q_m_ K_s_ 1/n R^2^	683.34 0.2982 0.4931 0.9934	637.31 0.2060 0.7445 0.9933	620.82 0.110 0.5451 0.9922	600.72 0.107 0.562 0.9913
Redlich-Peterson	$q_{e} = \frac{{A_{RP} C_{e} }}{{1 + B_{RP} C_{e}^{g} }}$	A_RP_ B_RP_ G R^2^	257.12 0.4515 0.9670 0.9901	202.32 0.3226 0.9984 0.9922	201.47 0.3711 0.9759 0.9912	175.75 0.3301 0.9786 0.9904

**Table 2:** Comparison of several adsorbents utilized to investigate VB adsorption from aqueous solution.

Adsorbent	Adsorbate	Adsorption Capacity (q_e_) mg g^−1^	References
Gellan gum/Arginine-bentonite	VB	322.58	^69^
Nanoporous calcined MCM-41 silica	VB	192.3	^70^
Incense stick ash	VB	105.57	^71^
Flower-shaped ZnO	VB	163	^72^
Carbon/Ba/alginate	VB	0.93	^73^
Fe_2_O_3_-activated Bakelite	VB	52.63	^74^
Low-cost activated carbon	VB	0.874	^75^
CS/In_2_S_3_	VB	683.34	This Work

#### Adsorption kinetics

**Figure 9:**
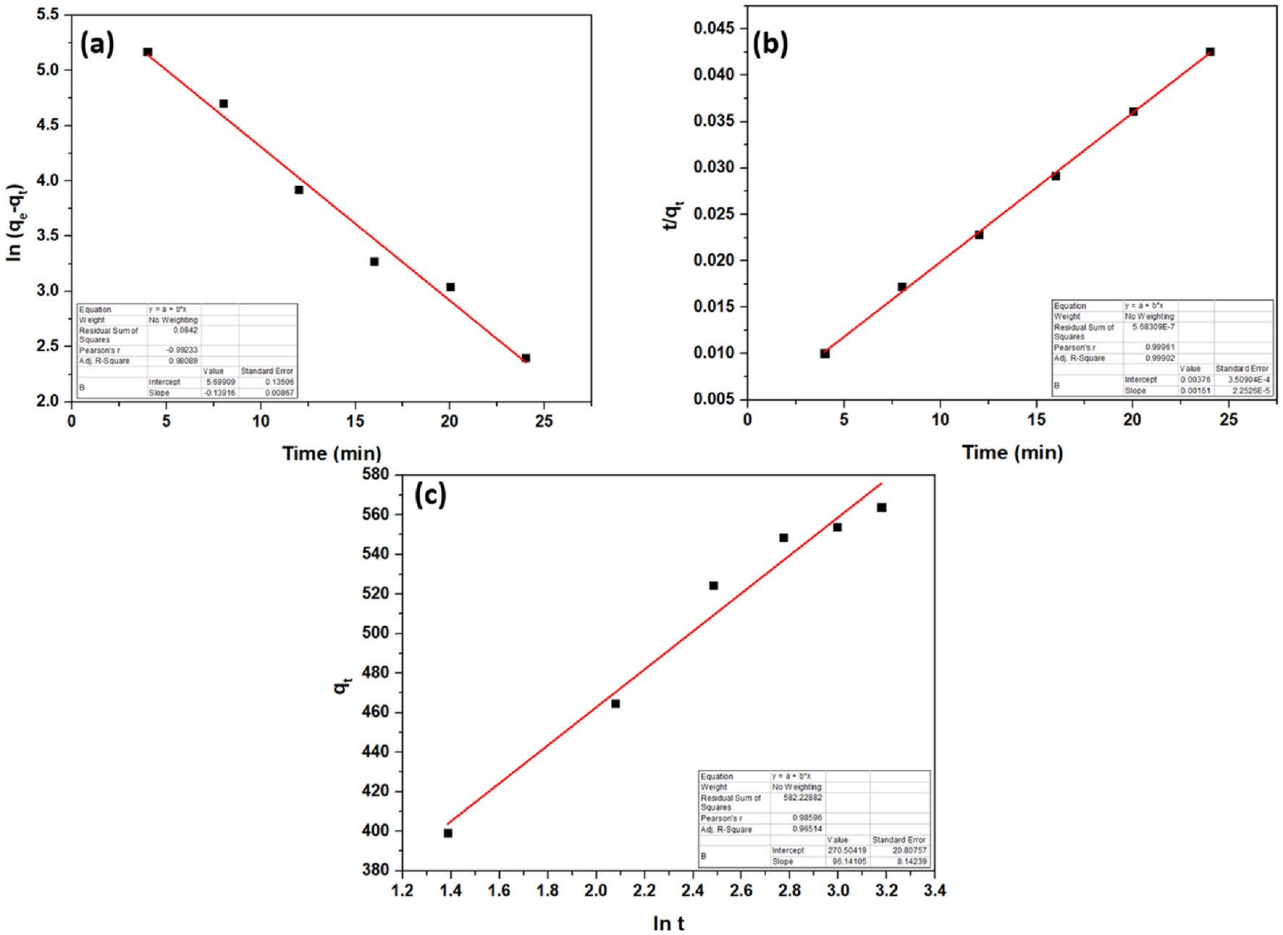
Plots showing (**a**) PFO, (**b**) PSO, and (**c**) Elovich kinetic models.

Adsorption efficiency depends on a fast reaction rate, a short contact time, and a significant adsorption capacity. The adsorption kinetics of VB by prepared CS/In_2_S_3_ nanocomposite was conducted using pseudo-first-order (PFO)^66^, PSO^67^, and Elovich kinetic models^68^, as shown in Fig. 9. The studies found that the adsorption followed PSO kinetics with a rate constant is 6.89 × 10^–4^ g mg^−1^ min^−1^ as it exhibited the maximum correlation coefficient (R^2^) of 0.999 (Table 3). This result indicates that the PSO kinetics fits the sorption behavior of the synthesized nanocomposite towards VB more than the PFO and Elovich models. The PSO model inferred chemisorption of the VB dye, which is backed by the isotherm studies as it obeyed the Sips isotherm model.

**Table 3:** Fitting of kinetic models.

Kinetic model	Linear equation	Correlation coefficient (R^2^)
PFO	$\ln (q_{e} - q_{t} ) = \ln q_{e} - k_{1} t$	0.9809
PSO	$\frac{t}{{q_{t} }} = \frac{1}{{k_{2} q_{e}^{2} }} + \frac{1}{{q_{e} }}t$	0.999
Elovich	$q_{t} = \frac{1}{\alpha }\ln (\alpha \beta ) + \frac{1}{\alpha }\ln t$	0.9651

#### Adsorption thermodynamics

Thermodynamic factors comprising Gibb's free energy (G°), enthalpy (H°), and entropy (ΔS°) are utilized for estimating the spontaneity level in a sorption technique because the decrease in G°, which has to be -ve for significant sorption to take place, indicates the degree of spontaneity^1,76^. Mathematically, G° and change in Gibbs free energy (ΔG°) are given in Eqs. (4) and (5), respectively.

4 $$ G^{0} = - RT\ln K_{ad} $$

5 $$ \Delta G^{0} = \Delta H^{0} - T\Delta S^{0} $$

where ΔH° and ΔS° are changes in enthalpy and entropy, respectively, and R, T, and K_ad_ are the gas constants, reaction temperature, and adsorption equilibrium constant. Additionally, K_ad_ is equivalent to C_ad_/C_e_, where C_e_ (mg L^−1^) is the equilibrium solute concentration and C_ad_ (mg L^−1^) is the concentration of the adsorbed solute^77^. Combining Eqs. (4) and (5), Vant Hoff's equation is obtained in Eq. (6).

6 $$ \ln K_{ad} = - \frac{{\Delta H^{0} }}{RT} + \frac{{\Delta S^{0} }}{R} $$

**Figure 10:**
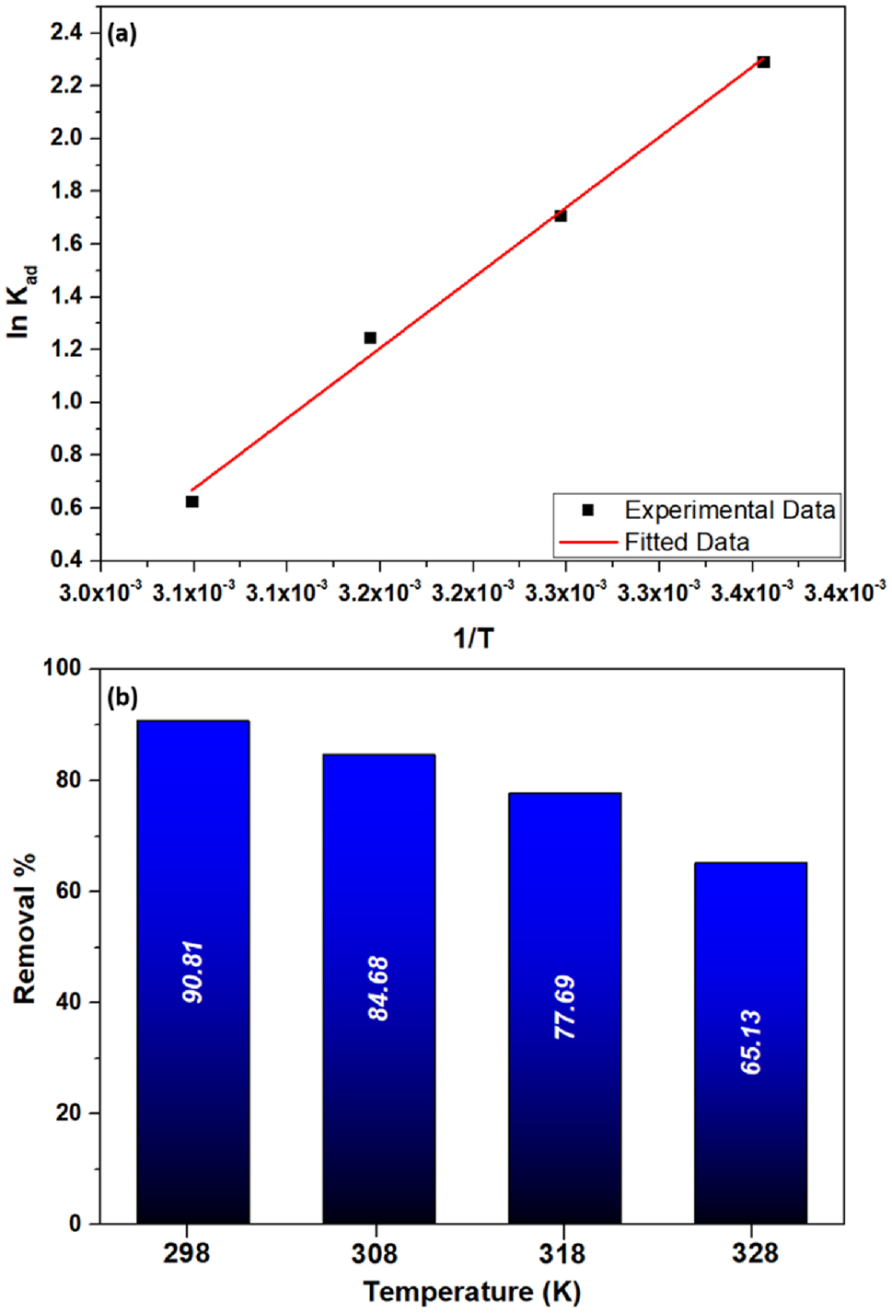
(**a**) Vant Hoff's plot for the removal of VB dye (**b**) Role of temperature on the adsorption of VB.

Plotting ln K_ad_ vs. 1/T yields the thermodynamic parameters for adsorption, as seen in Fig. 10a, and ΔH° and ΔS° are determined from the slope and intercept, correspondingly (Table 4). 10 mg of the composite was mixed with 100 ppm VB dye concentration and agitated for 60 min by varying temperature from 298 to 328 K. The obtained ΔH^0^ value is negative, which signifies exothermic adsorption, and the enthalpy value is −44.313 kJ mol^−1^. Negative ΔG^0^ values at all temperatures reflect the adsorption process's spontaneity and feasibility, and negative ΔS^0^ correlates to a reduction in the degree of freedom of the adsorbed substance^78^. Similar results were obtained by Thamer et al.^79^, where the negative ΔG^0^ value indicated spontaneous crystal violet adsorption on the ZVNi-NiO@Gr surface. Moreover, because desorption rises with temperature, as illustrated in Fig. 10b, the amount of VB adsorption reduces. Due to the weakening of bonds between the vicinal molecules and the active adsorbent, when the temperature rises, the adsorption may decrease.

**Table 4:** Thermodynamic variable at various temperatures.

Temperature (K)	ΔG^0^ (kJ mol^−1^)	ΔH^0^ (kJ mol^−1^)	ΔS^0^ (J K^−1^ mol^−^)
298	−5.673		
308	−4.376	−44.313	−129.53
318	−3.296		
328	−1.701		

#### Statistical physics modeling

**Scheme 1:**
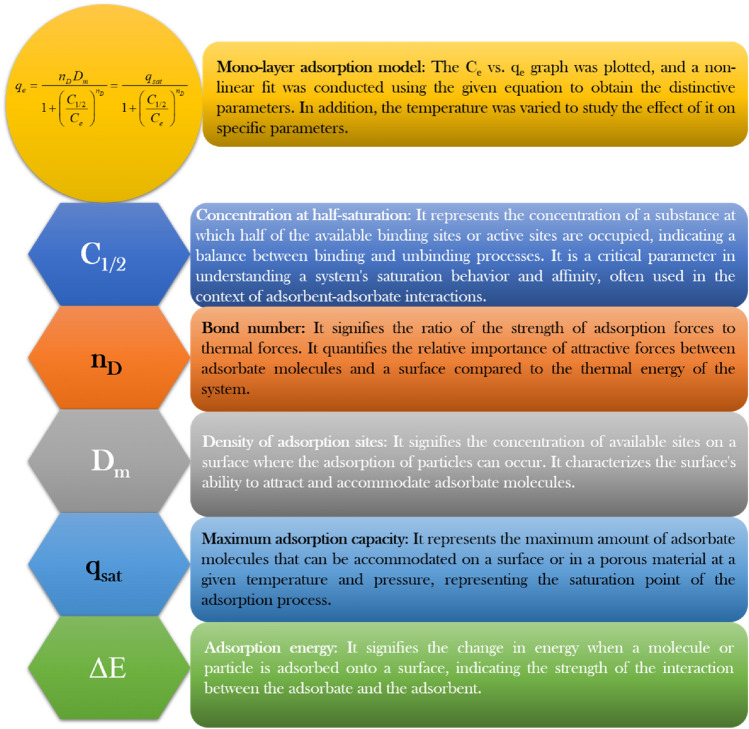
Significance of the terms obtained from SPM.

SPM was employed to analyze the adsorption mechanism and confirm the inference from isotherm studies and BET analysis. The modeling equations help to comprehend the adsorption of VB on CS/In_2_S_3_ using various parameters. Furthermore, the change in adsorption temperature also played a vital role, which can be confirmed through these modeling. Scheme 1 illustrates SPM analysis, highlighting the calculations and significance of each term. The expression for monolayer adsorption is shown below (Eq. 7)^80^; in this case, all the molecules have single adsorption energy^81^.

7 $$ q_{e} = \frac{{n_{D} D_{m} }}{{1 + \left( {\frac{{C_{1/2} }}{{C_{e} }}} \right)^{{n_{D} }} }} = \frac{{q_{sat} }}{{1 + \left( {\frac{{C_{1/2} }}{{C_{e} }}} \right)^{{n_{D} }} }} $$

This adsorption model has three variables. C_1/2_ is the concentration at half-saturation during adsorption, D_m_ is the density of adsorption sites, and n_D_ refers to the amount of VB molecules attached to specific adsorption sites. In this model, the active sites in CS/In_2_S_3_ were occupied entirely, forming a saturation point. The expression q_sat_ = n_D_*D_m_ refers to adsorption capacity at saturation point.

The expression for the double-layer adsorption model (Eq. 8) assumed that the adsorption of VB molecules on CS/In_2_S_3_ involved interaction between adsorbate and adsorbent and between the dye molecules^82^.

8 $$ q_{e} = n_{D} D_{m} \frac{{\left( {\frac{{C_{e} }}{{C_{1} }}} \right)^{{n_{D} }} + 2\left( {\frac{{C_{e} }}{{C_{2} }}} \right)^{{2n_{D} }} }}{{1 + \left( {\frac{{C_{e} }}{{C_{1} }}} \right)^{{n_{D} }} + \left( {\frac{{C_{e} }}{{C_{2} }}} \right)^{{2n_{D} }} }} $$

At half-saturation, C_1_ represents the concentration at which the initial layer forms, whereas C_2_ signifies the concentration during the second layer formation. Moreover, the saturated adsorption capacity is denoted as q_sat0_ = 2⋅n_D_*D_m_.

**Figure 11:**
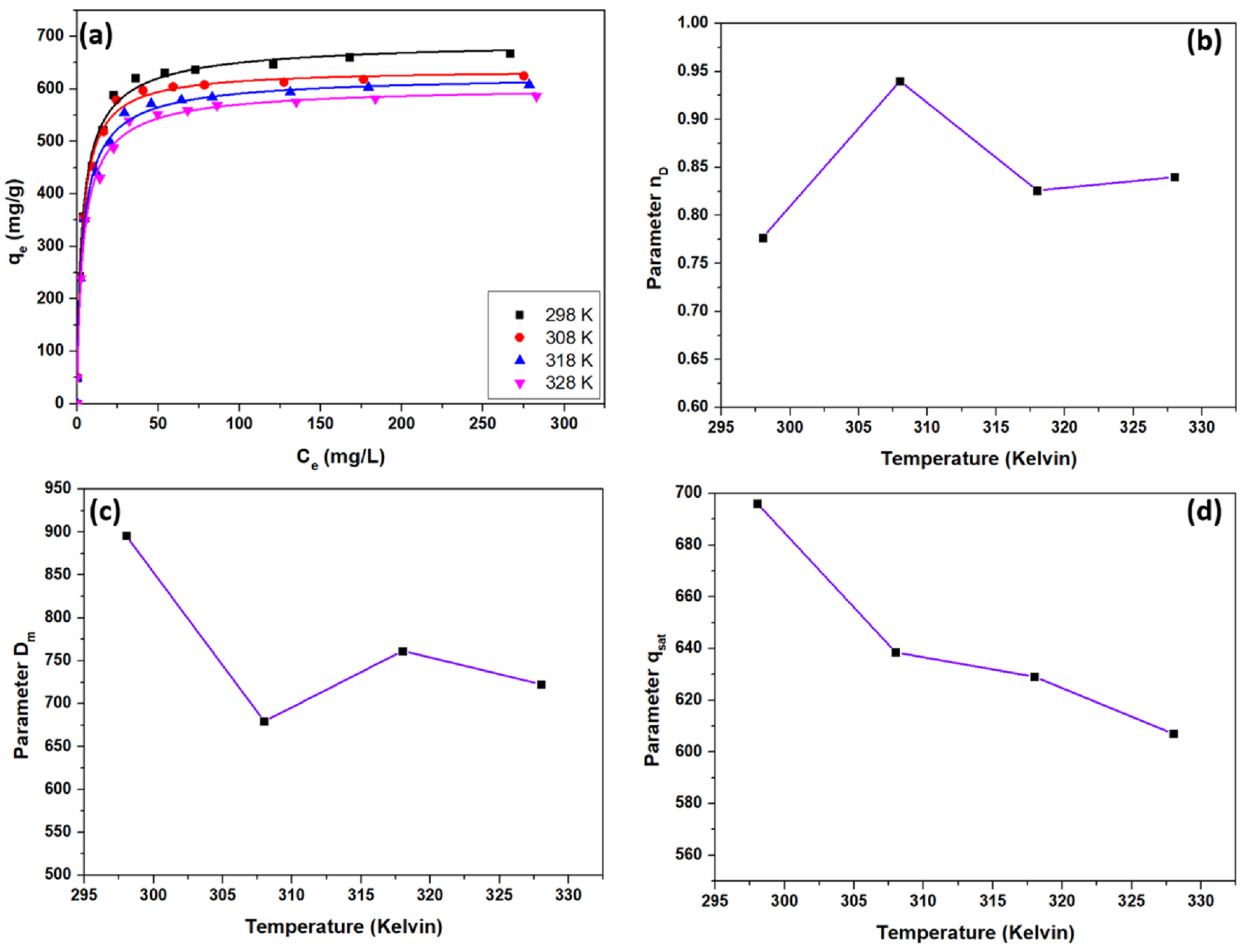
(**a**) Adsorption isotherm of VB on CS/In_2_S_3_ and their fitting to statistical physics model; Effect of temperatures on parameters (**b**) n_D_, (**c**) D_m_, and (**d**) q_sat_ for VB adsorption interpreted from the SPM.

In both the monolayer and double-layer adsorption models, the fitting results indicated a high degree of agreement with the investigational statistics, as the R^2^ values were close to 1. Nevertheless, this coefficient may not serve as the sole criteria for deciding on a model for determining VB's adsorption on CS/In_2_S_3_. The parameters obtained from the monolayer model were more practical and realistic compared to the double-layer model. So, to further investigate the dye’s adsorption mechanism on the fabricated nanocomposite, the statistical physics monolayer model was studied, as revealed in Fig. 11a. The fitted variables are discussed in Table 5.

**Table 5:** Adjusted parameters of the SPM for adsorption of VB on CS/In_2_S_3_.

Temperature (K)	n_D_	D_m_ (mg g^−1^)	q_sat_ (mg g^−1^)	C_1/2_ (mg L^−1^)	ΔE (kJ mol^−1^)	R^2^
298	0.777	896.03	696.19	3.36	−23.804	0.9921
308	0.94	679.26	638.50	3.15	−24.768	0.9924
318	0.826	761.58	629.06	3.58	−25.234	0.9919
328	0.840	722.61	606.99	3.88	−25.808	0.9917

#### *Bonded number of VB molecules (n*_*D*_*)*

The parameter n_D_ helps to interpret the steric hindrance by giving a theoretical concept for elucidation of the adsorption mechanism of VB on CS/In_2_S_3_^83^. Additionally, it highlights how much VB molecules have accumulated in the aqueous solution and how much of them have been adsorbed^84^. The adsorption parameter analyzes the adsorption orientation and is described using the following conditions:

The fact that the VB molecules link to mainly two sites of adsorption on CS/In_2_S_3_ and exhibit a pure parallel orientation indicates that the molecules of VB engage in a multi-interaction adsorption mode, according to condition I, where n_D_ < 0.5.

Condition II, where, n_D_ > 1, implies a multi-molecular adsorption process in which the VB dye molecules seek an active adsorption points and exhibit only non-parallel interactions.

The VB particles are implied to be sorbed in hybrid orientations, i.e., exhibiting mutually parallel and non-parallel interactions, by Condition III where 0.5 < n_D_ < 1.

According to the fitting parameters of the physical statistical method, as shown in Table 5, the adsorption of VB molecules followed a hybrid orientation, i.e., it obeyed both parallel and non-parallel interactions. It showcased multi-interaction and multi-molecular adsorption mechanisms as the n_D_ parameter values ranged from 0.777 to 0.94. The adsorption of AR1 dye on amphoteric adsorbent coating was reported by Azha et al.^85^, and the n_D_ parameters were found to be in the range of 0.52 to 0.83. It was inferred that the adsorption followed a multi-anchorage mechanism, similar to the present work. Furthermore, The fraction of parallel and non-parallel interaction during adsorption can be found by using a simple Eq. 9;

9 $$ n_{D} = x + (1 - x) \times 0.5 $$

Here, the n_D_ parameter value is obtained while fitting the SPM; x represents the fraction of non-parallel orientation, and 1-x represents the fraction of parallel orientation. At 298 K, the n_D_ parameter was found to be 0.777, and the obtained x value was 0.554, which implied that CS/In_2_S_3_ sorbed 55.4% of VB molecules through non-parallel interaction, whereas 44.6% of VB were adsorbed by parallel orientation. This result also coincides with the inference from isothermal studies and BET analysis. As the temperature increases, the n_D_ parameter values lie around 0.82 to 1. Temperature change has a frail impact on the molecule accumulation and succeeding anchorage on the adsorption site, as evidenced by this slight variation in the n_D_ trend (Fig. 11b)^86^. This inference overlaps with the q_m_ obtained from the Sips isotherm, as no significant decrease was observed.

#### *Density of adsorption sites present in CS/In*_*2*_*S*_*3*_* (D*_*m*_*)*

Figure 11c illustrates the disparity trend of parameter D_m_ with temperature for VB adsorption on CS/In_2_S_3_. According to the figure and Table 5, parameter D_m_ has a different variation trend with temperature than parameter n_D_, i.e., parameter D_m_ increases with temperature, while parameter n_D_ decreases, and vice-versa. The anchoring number (n_D_' = 1/n_D_) is known to be contrary to parameter n_D_, and reducing n_D_ induces a rise in n_D_', increasing adsorption density^87^. Additionally, the volume of operative adsorption sites on the adsorbent reduces as parameter n_D_ rises due to a reduced distance between adsorption sites on CS/In_2_S_3_^88^. In comparison to the work done by Sellaoui et al.^89^, the adsorption of VB on CS/In_2_S_3_ showed exothermic adsorption, while their research group inferred endothermic adsorption of nicotinamide and propranolol on magnetic-activated carbon. This led to the opposite trend in the D_m_ parameter as the initial decrease was attributed to the water adsorption, and a further increase was credited to the freely moving dye molecules due to the temperature rise^89^.

#### *Maximum adsorption capacity at saturation (q*_*sat*_*)*

The quantity of dye an adsorbent can adsorb at a saturated adsorption site is measured by its saturation adsorption capacity. The results in Table 5 indicate that VB has a very high saturation adsorption capacity. This conclusion can be attributed, among other things, to the size of the VB chemical structure, which affects the dye molecules' rate of agility, and the potential for a specific functional group interaction between dyes and adsorbents, which improves adsorption efficacy^37^. As demonstrated in Fig. 11d, as the temperature rises, the saturation adsorption capacity decreases, similar to data obtained in the thermodynamics analysis. This inference indicates that the adsorption mechanism follows the exothermic process. A similar hypothesis was postulated by Wang et al.^90^, where the adsorption of rhodamine B, congo red, and naproxen decreased with increased temperature.

#### Adsorption energy analysis

Calculating and studying adsorption energy can help understand how VB dye molecules attach to CS/In_2_S_3_. Table 5 displays the correlation findings for adsorption energy. The absorption energy was calculated via the concentration at half-saturation, as shown in Eq. 10.

10 $$ C_{1/2} = C_{s} e^{{\frac{\Delta E}{{RT}}}} $$

The half-saturation concentration is based on the dye's water solubility (Cs) and adsorption energy^83^. The SPM was used to fit experimental data, and the adsorption energies that define the interaction between VB molecules and the CS/In_2_S_3_ adsorbent surface were determined. As calculated, the negative adsorption energy values solidify the inference of exothermic adsorption processes. Moreover, the adsorption energies were close to the borderline value of 23 kJ mol^−1^, which signified the existence of both chemical and physical adsorption mechanisms. The chemical process was driven by covalent and ionic bonding, whereas the physical process was carried out due to Vander Waals interaction and hydrogen bonding^91^. From this, we can conclude that the SPM agrees with the isotherm studies, which suggest that the adsorption following the Sips isotherm model is an amalgamation of the Langmuir (chemisorption) and Freundlich (physisorption) isotherm model.

### Effect of different water samples

**Figure 12:**
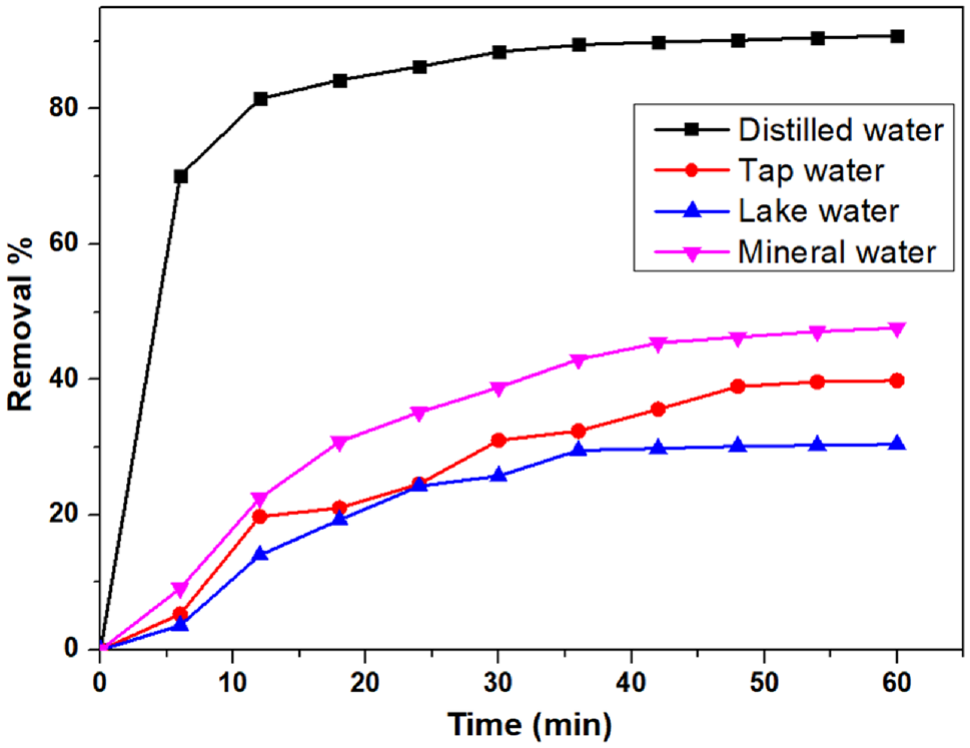
Effect of different water samples on the removal of VB.

The adsorption of VB was evaluated in four varied types of water matrices, as illustrated in Fig. 12. Mineral, tap, and lake water were the three kinds of water models collected, and they were equated with distilled water^92^. 50 mL of 100 ppm dye solution was made using the water samples, and 12 mg of nanocomposite was mixed and agitated for about 1 h. Out of the three, mineral water was the purest, showing a removal efficiency of 47.62%, whereas, in the case of tap water and lake water, the efficiency slumped up to 39.818% and 31.13%, respectively. Due to the presence of different inhibitory inorganic ions and organic substances in the previously mentioned water matrices at varied concentrations, the removal capacity of the adsorbent is usually decreased in these environmental waters. Because lake water contains more extra pollutants and surfactants than tap water, it may have been less effective as an adsorbent in the previous matrix.

### Effect of organic compounds

**Figure 13:**
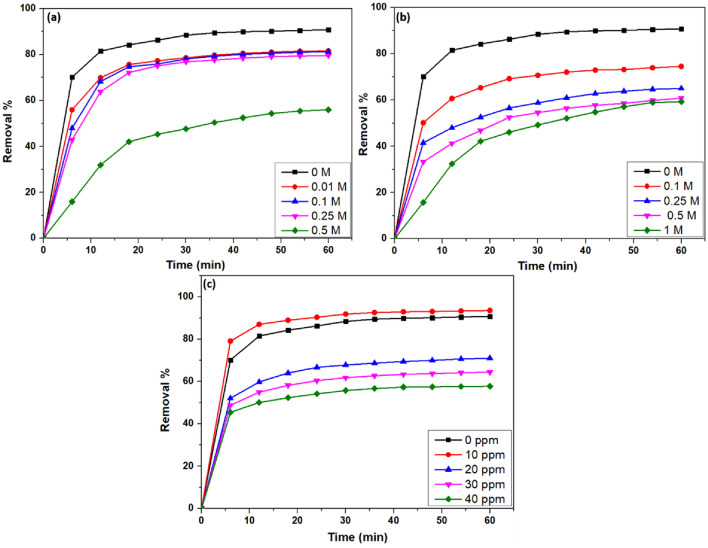
Impact of (**a**) acetone, (**b**) butanol, and (**c**) SDS on the removal of VB.

Studies explored the influence of organic compounds such as acetone, butanol, and sodium dodecyl sulfate (SDS) on the adsorption of VB dye by CS/In_2_S_3_ nanocomposite. Acetone is widely used in industry and everyday life because of its excellent solvent properties^93^. Butanol is used as a solvent for chemical reactions and is considered a potential gasoline replacement, whereas SDS is a surfactant and is commonly present in house cleansing products^94^. As illustrated in Fig. 13, 12  mg of nanocomposite was added to 100 ppm of VB dye, and the adsorption efficiency was investigated under different concentrations of organic compounds. Acetone and butanol, two commonly used solvents, exhibit a relatively strong quenching effect and have regeneration capabilities^95^. 1 M of acetone and butanol decreased the removal efficiency to 56.02% and 59.36%, respectively. The molecules of acetone and butanol competed with dye molecules for active surfaces as their increased concentration resulted in decreased dye adsorption. The dye sorption increased as the SDS surfactant concentration increased slightly (10 ppm)^96^. However, as the surfactant concentration increased further, the dye sorption was significantly suppressed by micelle formation and dye solubilization and was reduced to 58.61% at 40 ppm of SDS concentration.

### Effect of inorganic salts

**Figure 14:**
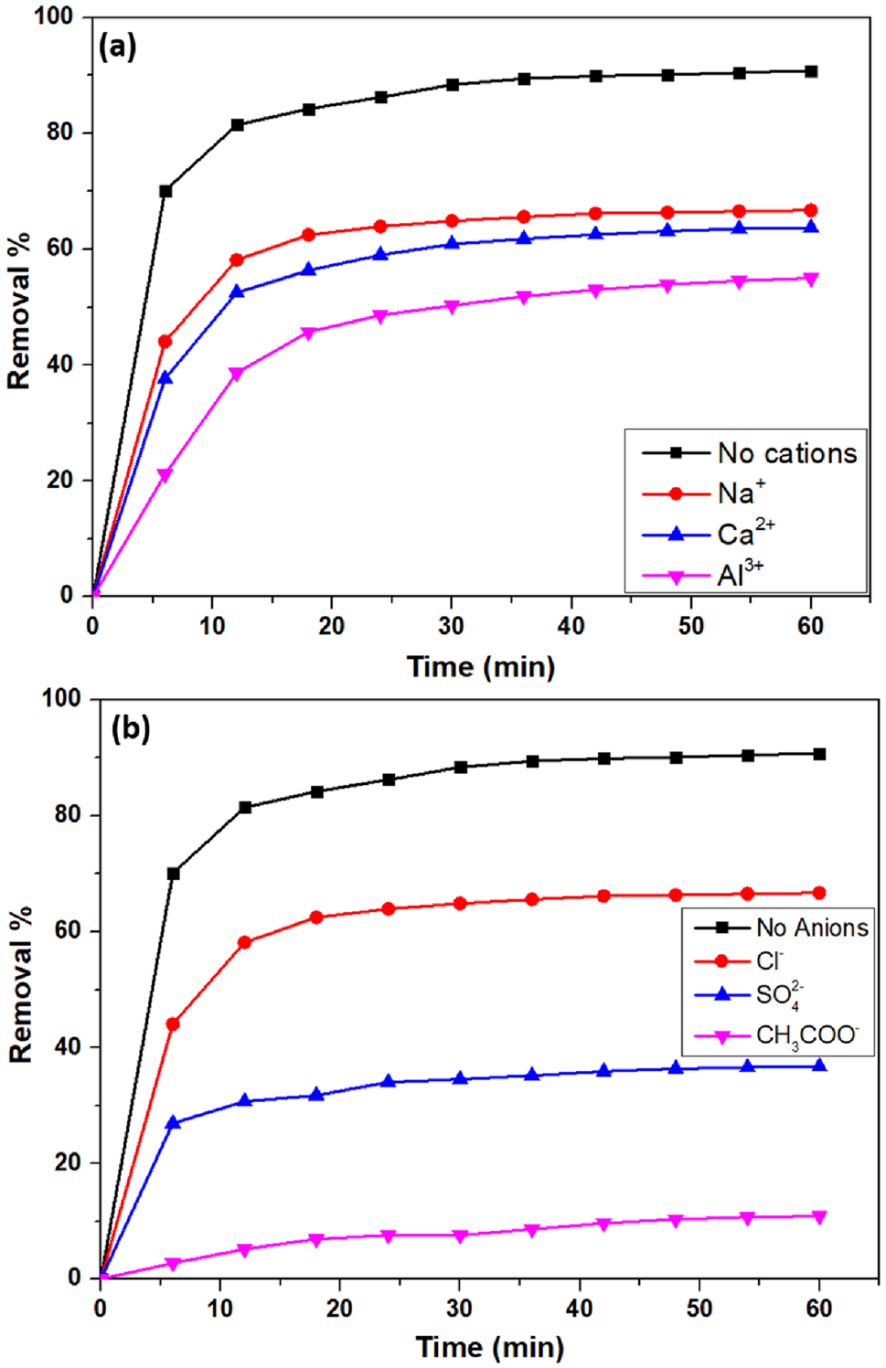
Role of (**a**) inorganic cations and (**b**) inorganic anions on the removal of VB.

The impact on the adsorption of dyes by inorganic salts has been examined in many research^97,98^. In this work, 0.01 M of salts such as NaCl, CaCl_2_, AlCl_3_, Na_2_SO_4_, and CH_3_COONa were introduced to 50 mL of 100 ppm VB dye to which 12 mg of nanocomposite was added to study the effect of cations (Fig. 14a) and anions (Fig. 14b). The addition of inorganic salts results in a reduction in adsorption efficacy as they might reduce the surface charge of the fabricated CS/In_2_S_3_ nanocomposite^99^. Due to the competition between ions formed by the hydrolysis of salts and organic molecules for adsorption sites, adsorption efficiency is slightly reduced^100^. In addition, these ions contest with VB molecules at adsorption sites, reducing VB's electrostatic attraction onto CS/In_2_S_3_ nanocomposite^101^. Due to inorganic cations, the removal efficiency slumped to 67.73, 63.72, and 55.14% for Na^+^, Ca^2+^, and Al^3+^, respectively, whereas for inorganic anions, the removal percentage decreased to 67.73, 36.81, and 10.95% for Cl^-^, SO_4_^2-^, and CH_3_COO^-^, respectively.

## Adsorption mechanism

**Figure 15:**
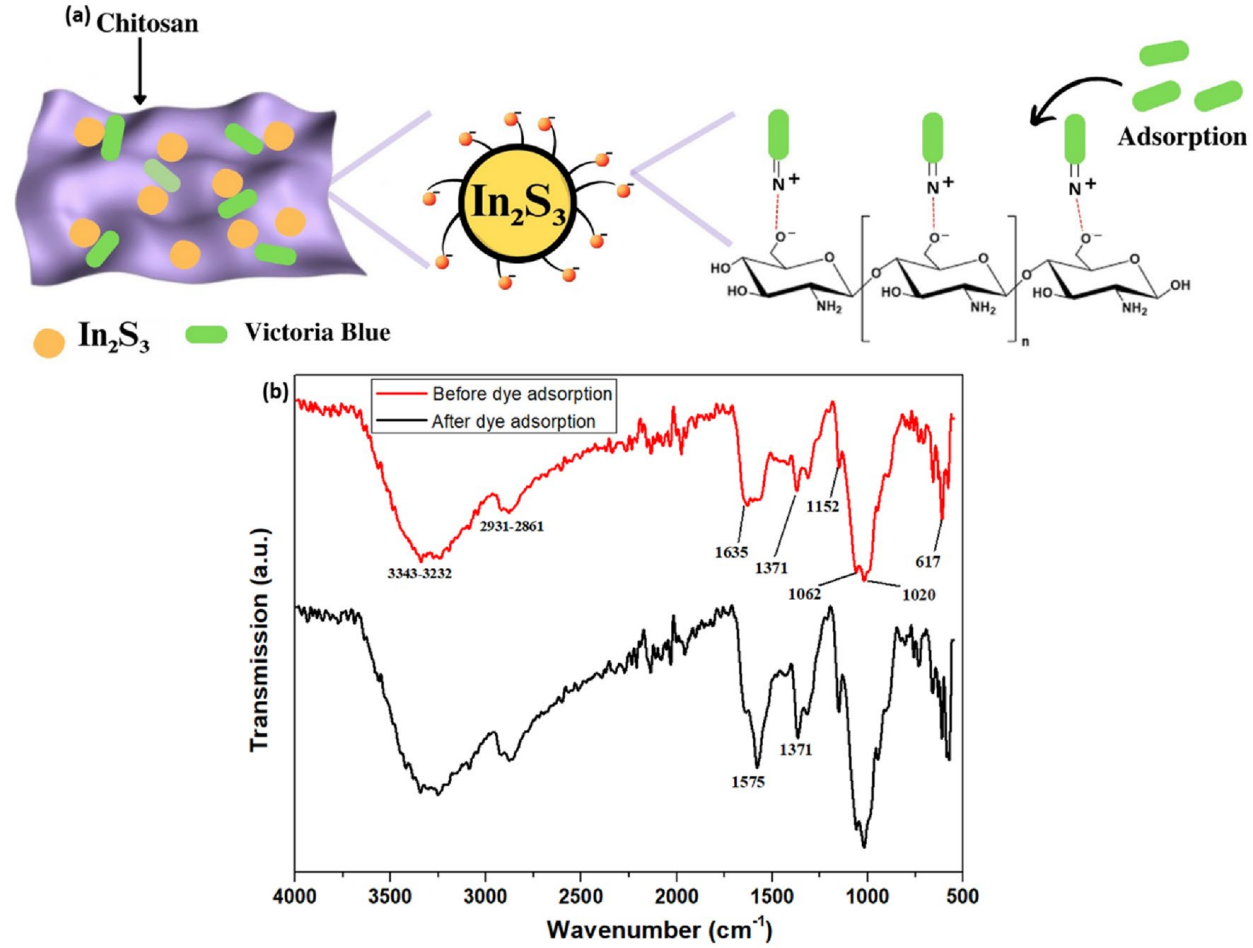
(**a**) Plausible mechanism of dye adsorption by fabricated CS/In_2_S_3_ adsorbent; (**b**) FTIR spectrum before and after adsorption.

The higher adsorption capacity of the CS/In_2_S_3_ nanocomposite for adsorbing VB can be credited to the -OH functional groups of chitosan (Fig. 15a), as its small surface area, high porosity, and high crystalline property increases its efficiency^102^. The functional groups in chitosan were assessed using FTIR spectroscopy to study the mechanism. The FTIR spectrum of CS/In_2_S_3_ nanocomposite is demonstrated in Fig. 15b. A high-intensity band at 3343–3232 cm^−1^ specifies N–H and O–H stretchings aroused due to hydrogen bonding in chitosan, overlying in the equivalent region. The 2931 cm^−1^ to 2861 cm^−1^ peaks designate symmetric and asymmetric stretching of C-H bonds. These bands are polysaccharide-specific and may be detected in the spectra of other polysaccharides such as xylan^103^, glucans^104^, and carrageenans^105^. The bands around 1635 cm^−1^ and 1590 cm^−1^ could be accredited to amide groups. Absorption bands at 1371 cm^−1^ and 617 cm^−1^ specify the vibration modes of the In-S band in In_2_S_3_^106^. A new strong peak appeared at 1575 cm^−1^ in the FTIR spectrum of CS/In_2_S_3_ after 5 cycles and can be allocated to N–H bending of a primary amine in chitosan overlapping with N–H group of adsorbed VB dye on chitosan surface^107^. A small peak around 880 cm^−1^ could be due to the plane bending of C-H in the polysaccharide ring. The increased intensity of the band at 1371 cm^−1^, which is the distinguishing stretching of the CH_3_ stretching, could be due to the improved number of CH_3_ groups from the adsorbed VB molecules^108^. An absorption band could confirm the asymmetric stretching of the C–O–C group at 1152 cm^−1^. The bands situated at 1062 cm^−1^ and 1020 cm^−1^ correspond to the stretching of C-O bonds. All of these bands correlate well with the FTIR spectra of chitosan that were previously published^109,110^. The peaks around 3360 cm^−1^ (N–H),1640 cm^−1^ (amide I), 1148 cm^−1^ (C–N), and 1020 cm^−1^ (C–O) imply that the adsorption of dye was onto the hydroxyl groups of chitosan via covalent bonding^111^.

## Reusability analysis

**Figure 16:**
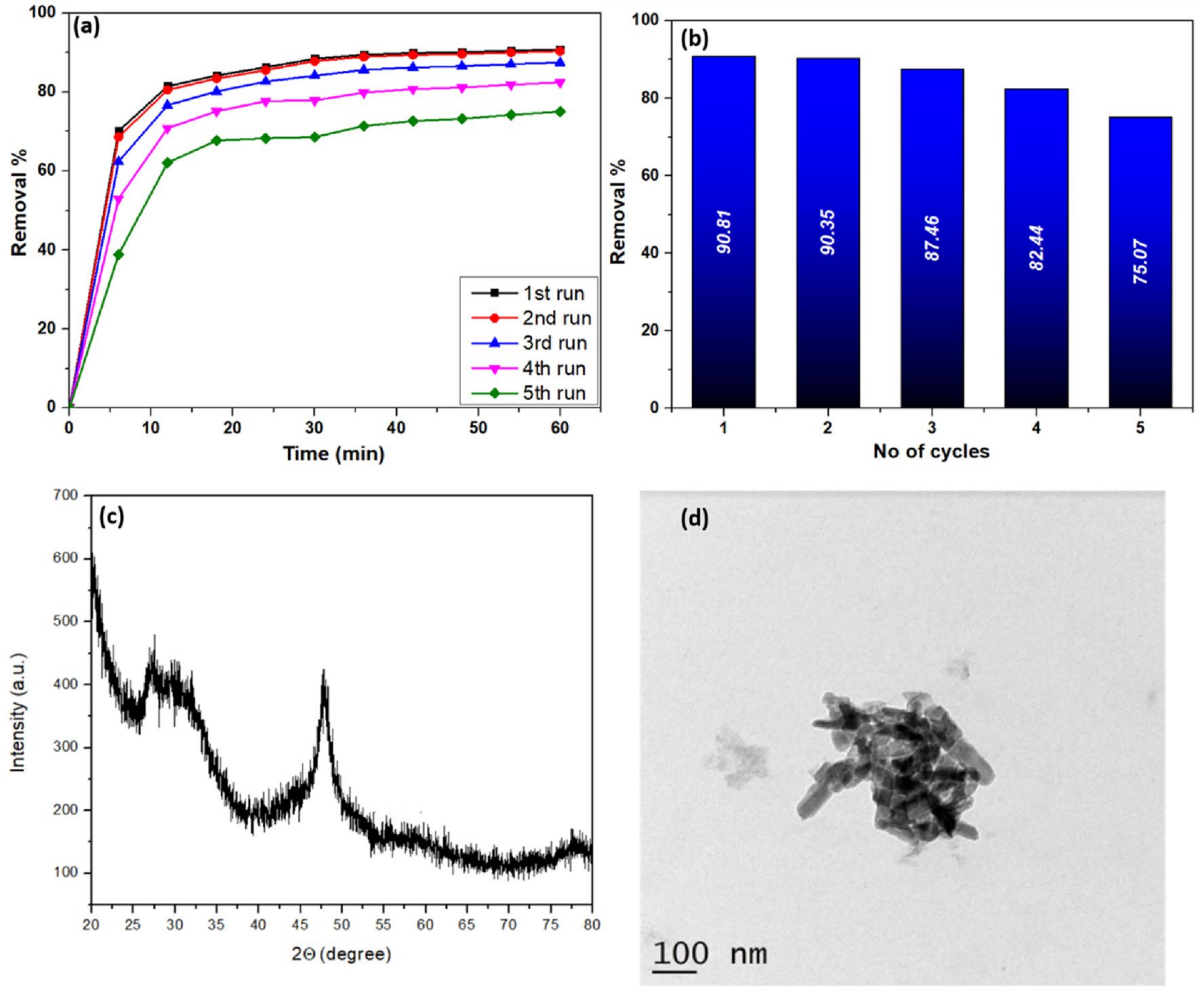
(**a**) Efficiency of VB removal, (**b**) Graph showing removal percentage during various cycles, (**c**) XRD plot, and (**d**) TEM image after dye adsorption.

As it is imperative to assess the efficacy of the adsorbent from a re-application standpoint and from an economic perspective, the reusability of the adsorbent is analyzed (Fig. 16a). The dye was regenerated using acetone as a desorption media. 20 mL of acetone was added to the filtered nanocomposite, and then it was thoroughly shaken for 30 min, and the supernatant was centrifuged. Further, the derived nanocomposite was cleaned with de-ionized water and desiccated for further adsorption analysis for five cycles. The 2nd run showed an efficiency of 90.35%, while the removal efficiency slumped to 75.07% during the 5th cycle, as shown in Fig. 16b. This reduction in the removal ability in the 5th cycle may be due to the disintegration of the active sites. Moreover, no significant change in the FTIR bands, as shown in Fig. 15b, indicates the high stability and reusability of the prepared adsorbent after five cycles. In addition, the XRD plot (Fig. 16c) and TEM image (Fig. 16d) of the nanocomposite after five cycles confirm the constancy of the fabricated nanocomposite.

## Conclusion

CS/In_2_S_3_ nanocomposite was manufactured by a facile co-precipitation method. The adsorption studies were conducted to understand the various aspects influencing the removal performance, for example, the initial concentration of the dye, adsorbent dosage, and contact time. The effect of temperature and thermodynamic studies is also carried out, along with the impact of various organic compounds, inorganic salts, and water matrices on VB adsorption. The nanocomposite was characterized to assign its structure, texture, morphology, and chemical composition. XRD, TEM-SAED, SEM, EDAX, XPS, Elemental Mapping, FTIR, and BET analyses were conducted to characterize the nanocomposite. The HR-TEM images show the accumulation of the In_2_S_3_ nanoparticles on the CS polymer matrix. The nanocomposite showed an appreciable q_m_ of 683.34 mg g^−1^ and an excellent removal efficiency of 90.81% for the 100 ppm VB dye removal using 12 mg (0.24 g L^−1^) of the nanocomposite. The BET isotherm of the nanohybrid showed type I hysteresis and type IV adsorption curves. Also, the adsorption performance is defined by the Sips adsorption isotherm and PSO kinetics having the value of rate constant k_2_ of 6.357 × 10^–3^ g mg^−1^ min^−1^. The sips adsorption isotherm model was a combination of Langmuir and Freundlich's adsorption isotherm model, and the adsorption mechanism was further investigated using SPM. The SPM interpreted the bonded number of dye molecules, the density of adsorption sites, the saturation adsorption capacity of adsorbent, and adsorption energies, which helped in understanding the mechanism of adsorption of VB on fabricated novel CS/In_2_S_3_ nanocomposite. From this, it can be inferred that the nanocomposite adsorbed the dye molecules in a mixed orientation involving multi-molecular and multi-interaction mechanisms. Moreover, the nanocomposite showed excellent stability and was reusable up to 5 times.

## Data Availability

All data generated or analyzed during this study are included in this published article.
